# Structural diversity and therapeutic potential of phenanthrenes from the *Dendrobium* genus: a comprehensive review (1987–2025)

**DOI:** 10.3389/fphar.2026.1800915

**Published:** 2026-05-15

**Authors:** Jie Jian, Junhao Wang, Sijia Wu, Wangya Jia, Wenxu Lu, Zixu Xu, Aizhen Xiong, Li Yang, Hong Xu

**Affiliations:** The MOE Key Laboratory for Standardization of Chinese Medicines, The SATCM Key Laboratory for New Resources and Quality Evaluation of Chinese Medicines, Institute of Chinese Materia Medica, Shanghai University of Traditional Chinese Medicine, Shanghai, China

**Keywords:** anti-inflammatory activity, antitumor activity, *Dendrobium*, metabolic diseases, natural products, phenanthrenes, structural diversity, traditional Chinese medicine

## Abstract

For millennia, *Dendrobium* Sw., the second-largest genus of Orchidaceae, has been widely applied in traditional Chinese medicine to nourish yin, clear heat, and promote body fluid. Its stems are a rich source of phenanthrenes, which are low molecular weight polycyclic aromatic metabolites with remarkable structural diversity and broad-spectrum bioactivities. This review provides the first comprehensive and systematic analysis of 158 naturally occurring phenanthrenes isolated from 53 *Dendrobium* taxa (51 species, 1 variety, and 1 horticultural cultivar) based on an extensive literature survey of international and Chinese databases (PubMed, Web of Science, SciFinder, CNKI, etc.) from 1987 to 2025. The article summarizes pharmacopoeial records, classical texts, and theses. The identified metabolites include simple phenanthrenes, 9,10-dihydrophenanthrenes, diphenanthrene dimers, phenanthrenequinones, and other phenanthrene derivatives, often functionalized with hydroxyl, methoxyl, and carboxyl functionalities. Among 158 phenanthrene metabolites isolated from *Dendrobium* species, 64 compounds (40.5%) have been evaluated in at least one bioactivity assay, with reported activities spanning anti-tumor (cytotoxic), anti-inflammatory, antioxidant, antidiabetic, anti-fibrotic, antiplatelet, or antimicrobial endpoints. However, the majority (54/64, 84.4%) are supported solely by *in vitro* screening data (IC_50_ < 100 μM in cell-based assays), while only 8 compounds (12.5%) have validated mechanisms and a mere 4 compounds (6.2%) demonstrate *in vivo* efficacy in animal models. This quantitative summary reflects research intensity rather than therapeutic potential, given significant heterogeneity in assay systems and the absence of standardized activity thresholds across studies. Mechanistic studies have revealed that these compounds modulate key signaling. thereby offering therapeutic potential against cancer, diabetes mellitus, metabolic dysfunction-associated fatty liver disease (MAFLD), osteoarthritis, and thrombosis. Despite the bioactivity profile, clinical translation is limited by *in vivo* validation and the lack of pharmacokinetic data. Future studies should focus on systematic preclinical evaluation. Multi-omics mechanistic studies are needed to advance these natural scaffolds into clinically viable therapeutics.

## 1 Introduction

For over two millennia, *Dendrobium* Sw., as the second-largest genus in Orchidaceae, has been employed in traditional Chinese medicine (TCM) to nourish yin, clear heat, and generate body fluid (Zhang et al., 2003). Comprising more than 1,500 species distributed across tropical and subtropical Asia, Australia, and the Pacific Islands, this genus includes over fifty species officially documented in the *Chinese Pharmacopoeia* or used regionally as sources of the traditional remedy “shi-Hu” (Ji, 1999, Hou et al., 2012, Li et al., 2023; Chinese Pharmacopoeia Commission, 2020). Ancient texts, including *Shen Nong’s Herbal Classic* (∼200 BCE), documented the efficacy of this genus in alleviating thirst, throat inflammation, and gastric discomfort (Lin et al., 2003; Zhang et al., 2003; Zheng et al., 2018), while modern clinical practice has extended its use as an adjunct treatment for diabetes, cancer, and chronic inflammatory disorders (Cheng and Guo, 2001; Zhou et al., 2022).

The therapeutic properties of *Dendrobium* are increasingly attributed to its distinctive repertoire of small-molecule metabolites, particularly phenanthrenes—a rare class of polycyclic aromatic compounds that serve as chemotaxonomic markers and principal bioactive metabolites (Cheng and Guo, 2001; Shen et al., 2022; Li et al., 2023). Although polysaccharides, alkaloids, bibenzyls, sesquiterpenoids, lignans, and flavonoids are also present, phenanthrenes exhibit high abundance, structural diversity, and broad-spectrum pharmacological activities, including antitumor, antidiabetic, anti-inflammatory, antioxidant, antifibrotic, and antiplatelet properties (Liu et al., 2025).

Over the past four decades, phytochemical investigations have revealed an exceptionally rich array of phenanthrenes in *Dendrobium* species, encompassing simple phenanthrenes, 9,10-dihydrophenanthrenes, diphenanthrene dimers, phenanthrenequinones, and various phenanthrene derivatives. Nevertheless, existing reviews on *Dendrobium* phenanthrenes remain fragmented and structurally incomplete, exhibiting three major limitations that the present work directly addresses. First, previous compilations typically covered merely 34–81 compounds (Teixeira da Silva and Ng, 2017; Zhao et al., 2022; Li et al., 2023), or focused exclusively on several representative phenanthrene metabolites (Wei et al., 2024), accounting for less than 40% of the currently known chemical space. Although Zhai et al. (2022) substantially expanded this coverage by summarizing 124 phenanthrenes from 44 *Dendrobium* species within their broader review of natural stilbenes, their classification scheme was oversimplified, categorizing phenanthrenes into only three groups—simple phenanthrenes, dihydrophenanthrenes, and other phenanthrenes—without adequately reflecting structural diversity. Furthermore, neither this nor previous reviews have systematically integrated comprehensive structural classification, species distribution, in-depth molecular mechanisms, structure–activity relationships, and translational bottlenecks, resulting in a significant disconnect between compound cataloguing and biological interpretation that has impeded rational drug discovery.

To overcome these limitations, the present review represents the first systematic analysis unifying four previously disconnected dimensions of *Dendrobium* phenanthrene research. Regarding chemical structural classification, we have catalogued 158 naturally occurring phenanthrene metabolites from 53 *Dendrobium* taxa (51 species, 1 variety, and 1 horticultural cultivar) species, representing a 27% expansion beyond the most comprehensive previous compilation (Zhai et al., 2022). These metabolites are systematically classified into seven categories based on parent nucleus and substitution patterns: simple phenanthrenes, 9,10-dihydrophenanthrenes, diphenanthrene dimers, phenanthrenequinones, phenanthrene glycosides, spiro-phenanthrenes, phenanthrene-bibenzyl heterodimers, and phenanthrenederivatives, thereby enabling in-depth structure–activity relationship analysis. Concerning literature integration, we have synthesized global and Chinese literature from 1958 to 2025, including classical TCM texts, pharmacopoeial records, and a limited number of phenanthrenes reported in Chinese dissertations with verified primary literature support, thereby capturing the complete discovery timeline (Cai et al., 2020), particularly the continuous expansion of dimers and complex oligomeric phenanthrenes since 2016, and identifying phenanthrene metabolites in an expanded range of *Dendrobium* species. With respect to bioactivity and mechanistic elucidation, beyond structural cataloguing, we have comparatively analyzed sporadically reported *in vitro* and *in vivo* bioactivities, identifying shared molecular targets that account for the polypharmacological characteristics of this compound class. Regarding translational applications, we have explicitly delineated critical bottlenecks in pharmacological efficacy and clinical pharmacokinetics that impede bench-to-bedside translation, and performed pharmacokinetic and drug-likeness predictions for three compounds with validated *in vivo* activity, providing a foundation for industrial translation. Collectively, through the integration of these dimensions, this review transforms fragmented *Dendrobium* phenanthrene literature into a coherent scaffold library with mechanistic and translational annotations, offering a fundamentally distinct resource from previous compilations and establishing a foundation for modern drug discovery.

## 2 Structure diversity of *Dendrobium* phenanthrenes

**FIGURE 1:**
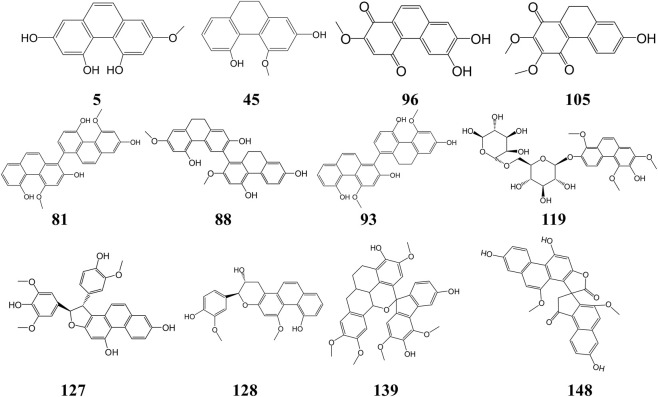
Chemical structures of representative simple phenanthrenes (5), simple 9,10-dihydrophenanthrenes (45), phenanthrene dimer (81), diphenanthrene dimer (88), phenanthrene-diphenanthrene dimer (93), phenanthrenequinones (96,105), phenanthrene glycosides (119), phenanthrene heterodimers (127,128,139) spiro-phenanthrenes (148) isolated from the *Dendrobium* plants.

Phenanthrenes are polycyclic aromatic hydrocarbons composed of three fused benzene rings. Although rare in the plant kingdom, they occur consistently in selected families, such as Orchidaceae, Juncaceae, Annonaceae, Aristolochiaceae, Cannabaceae, Combretaceae, Dioscoreaceae, Euphorbiaceae, Lauraceae, Malpighiaceae, and Stemonaceae. Within the Orchidaceae family, the most variable repertoire is observed in *Dendrobium*, *Pholidota*, *Bulbophyllum,* and *Bletilla*, with *Dendrobium* exhibiting the greatest structural variability (Adriána et al., 2008; Antonino et al., 2022; Tóth et al., 2018). To comprehensively summarise the names and structures of *Dendrobium* phenanthrenes reported to date, we conducted a multi-tiered literature search across nine electronic databases spanning chemistry, pharmacology and ethnobotany: PubMed, Web of Science, SciFinder, Scopus, CNKI, Wanfang, VIP, the Chinese Pharmacopoeia (2025 edition) and classical TCM texts (*Shen Nong’s Herbal Classic*). The search employed name-based keywords (*Dendrobium*, *shi-hu*, 石斛) combined with phenanthrene structural classifiers. Each retrieved metabolite was assigned a unique identifier and categorised by core skeleton: simple phenanthrenes (C-9/C-10 unsaturated), 9,10-dihydrophenanthrenes (saturated), phenanthrenequinones (ortho- or para-diketone), diphenanthrene dimers (C–C or C–O–C linked), phenanthrene glycosides (4-O-β-D-Glc, 4-O-β-D-Xyl, 5-O-β-D-Glc), spiro-phenanthrenes (cyclopentane-fused), phenanthrene lactones (five/six-membered ring-fused), phenanthrene heterodimers (cross-coupled with fluorene, pyrrole, tetrahydropyran, dihydrofuran) and (Figure 1). Hydroxy, methoxy and carboxyl substituents were precisely mapped to positions C-1–C-10, with stereochemistry annotated where applicable (e.g., 9S-hydroxy), and species-level occurrence frequencies were recorded. Ambiguous analogues were cross-verified via SciFinder and PubChem CID to eliminate redundancies, ensuring the final compilation reflects 158 unique, unambiguously characterised phenanthrene metabolites from 53 *Dendrobium* taxa (51 species, 1 variety, and 1 horticultural cultivar). The principal structural classes of phenanthrene compounds from the genus *Dendrobium* are shown in Figure 1.

### 2.1 Simple phenanthrenes

**FIGURE 2:**
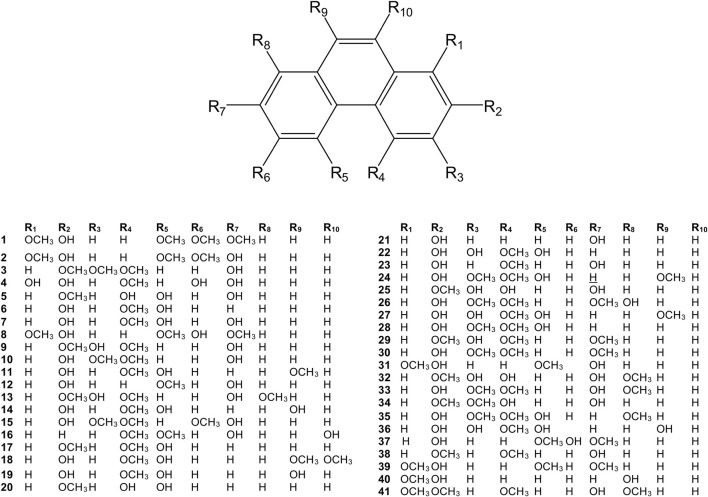
Chemical structures of simple phenanthrenes (1–41) isolated from the *Dendrobium* plants.

Quantitative analysis of 41 simple phenanthrenes (1–41) from 33 *Dendrobium* species reveals probabilistic substitution patterns that reflect enzymatic regioselectivity in biosynthesis (Figure 2; Table 1). C-2 hydroxylation is near-universal, occurring in 65% (27/41) of compounds, establishing this position as a conserved biosynthetic anchor. C-4 methoxylation predominates at 65% (27/41), while methoxylation at C-2 is less frequent. Additional hydroxylation shows broader distribution: C-5 and C-7 positions are hydroxylated in approximately 37% (15/41) and 48% (20/41) of compounds, respectively. Carboxyl groups remain sparsely distributed (5%, 2/41), with only one compound (21) bearing exclusively hydroxyl substituents. Notably, C-10 substitution is exceedingly rare (4.9%, 2/41), documented only in plicatol A (18) from *D. plicatile* and *D. nobile* (Honda and Yamaki, 2000; Yang et al., 2007).

**TABLE 1:** Forty-one simple phenanthrene (1–41) and their distribution in *Dendrobium* plants.

No.	Common name or/and IUPAC systematic nomenclature	Origin	References
1	chrysotoxene (2- hydroxy-1,5,6,7-tetramethylphenanthrene; 1,5,6,7-tetramethoxy-2-hydroxyphenanthrenol)	*D. chrysotoxum*	Ma et al. (1994), Hu et al. (2012), Liu et al. (2022), Yang et al. (2001)
2	confusarin (2,7-dihydroxy-1,5,6-trimethoxyphenanthrene)	*D. chrysotoxum*	Ma et al. (1994), Yang et al. (2001), Hu et al. (2012), Liu et al. (2022)
		*D. chryseum*	Ma et al. (1998)
		*D. fimbriatum*	Bi et al. (2003)
		*D. nobile*	Zhang et al. (2008), Zhou et al. (2016), Xu et al. (2018), Zhang et al. (2019)
		*D. officinale*	Zhao et al. (2018), Cui et al. (2019)
		^a^ *D. Sonia*	Cai (2020) ^b^
		*D. thyrsiflorum*	Li et al. (2016)
		*D. aphyllum*	Zhao et al. (2024)
3	7-hydroxy-2,3,4-trimethoxyphenanthrene	*D. chrysotoxum*	Liu et al. (2022)
4	1,2,6,7-tetrahydroxy-4-methoxyphenanthrene	*D. chrysotoxum*	Liu et al. (2022)
5	lusianthrin (4,5,7-trihydroxy-2-methoxyphenanthrene)	*D. nobile*	Zhou et al. (2016)
		^a^ *D. Sonia*	Cai (2020) ^b^
6	moscatin (plicatol B; 2,5-dihydroxy-4-methoxyphenanthrene)	*D. aphyllum*	Chen et al. (2008), Zhang et al. (2008), Yang et al. (2015)
		^c^ *D. aurantiacum* var. *denneanum*	Liu et al. (2009)
		*D. chrysotoxum*	Li et al. (2009), Hu et al. (2012), Weng et al. (2017), Liu et al. (2022)
		*D. chryseum*	Zheng et al. (2000)
		*D. chrysanthum*	Yang et al. (2006)
		*D. denneanum*	Lin et al. (2013)
		*D. densiflorum*	Fan et al. (2001)
		*D. devonianum*	Wu et al. (2019)
		*D. fimbriatum*	Xu et al. (2014)
		^d^ *D. hongdie*	Chen et al. (2015)
		*D. hancockii*	Shang et al. (2019)
		*D. loddigesii*	Chen et al. (1994), Ito et al. (2010), Li et al. (2013)
		*D. moschatum*	Yang et al. (2022)
		*D. nobile*	Zhou et al. (2016), Zhou et al. (2018)
		*D. officinale*	Cao et al. (2019), Cui et al. (2019), Zhao et al. (2018)
		*D. rotundatum*	Majumder and Sen (1987b), Majumder et al. (1992)
		*D. plicatile*	Honda and Yamaki (2000)
		*D. primulinum*	Ye et al. (2016)
		*D. senile*	Phyu et al. (2022)
		^a^ *D. Sonia*	Cai (2020) ^b^
		*D. thyrsiflorum*	Zhang et al. (2004), Zhang et al. (2005)
		*D. trigonopus*	Hu et al. (2008)
		*D. wardianum*	Zhang et al. (2017)
		*D. polyanthum*	Hu et al. (2009)
7	2,5,7-trihydroxy-4-methoxyphenanthrene	*D. senile*	Phyu et al. (2022)
8	denthyrsinin (2,6-dihydroxy-1,5,7-trimethoxyphenanthrene; 1,5,7-trimethoxyphenanthrene-2,6-diol)	*D. densiflorum*	Fan et al. (2001)
		*D. palpebrae*	Kyokong et al. (2019)
		*D. plicatile*	Chen et al. (2020)
		*D. thyrsiflorum*	Zhang et al. (2005)
9	epheranthol B (3,7-dihydroxy-2,4-dimethoxyphenanthrene)	*D. chrysotoxum*	Hu et al. (2012)
			Li et al. (2009)
		*D. hainanense*	Zhang et al. (2015)
		*D. nobile*	Zhang et al. (2008)
		*D. plicatile*	Yamaki and Honda (1996)
			Chen et al. (2020)
		^a^ *D. Sonia*	Cai (2020) ^b^
		*D. thyrsiflorum*	Li et al. (2016)
		*D. aphyllum*	Zhao et al. (2024)
10	Nudol (2,7-dihydroxy-3,4-dimethoxyphenanthrene)	*D. hainanense*	Zhang et al. (2015)
		*D. nobile*	Zhou et al. (2016)
		*D. officinale*	Cui et al. (2019)
		*D. plicatile*	Chen et al. (2020)
		*D. rotundatum*	Majumder et al. (1992)
		^a^ *D. Sonia*	Cai (2020) ^b^
		*D. aphyllum*	Zhao et al. (2024)
11	2,5-dihydroxy-4,9-dimethoxyphenanthrene (4,9-dimethoxyphenanthrene-2,5-diol)	*D. chrysotoxum*	Li et al. (2009)
			Weng et al. (2017)
			Liu et al. (2022)
		*D. chrysanthum*	Yang et al. (2006)
		*D. hancockii*	Shang et al. (2019)
		*D. nobile*	Yang et al. (2007), Zhang et al. (2008)
		*D. palpebrae*	Kyokong et al. (2019)
		*D. senile*	Phyu et al. (2022)
		^a^ *D. Sonia*	Cai (2020) ^b^
		*D. speciosum*	Takamiya et al. (2019)
		*D. spectabile*	Takamiya et al. (2019)
		*D. thyrsiflorum*	Li et al. (2016)
​	​	*D*. *stuposum*	Qin et al. (2019)
12	Flavanthrinin (2,7-dihydroxy-5-methoxyphenanthrene)	*D. brymerianum*	Klongkumnuankarn et al. (2015)
		*D. lindleyi*	Qin et al. (2022)
		*D. nobile*	Zhang et al. (2008)
		*D.venustum*	Sukphana et al. (2014)
13	2,4,8-trimethoxy-phenanthrene-3,7-diol	*D.crumenatum*	Kongkatitham et al. (2023)
		*D. loddigesii*	Ito et al. (2010)
		^a^ *D. Sonia*	Cai (2020) ^b^
14	Rotundatin (2,5,9-dihydroxy-4-methoxyphenanthrene]	*D. loddigesii*	Ito et al. (2010)
		*D. rotundatum*	Majumder et al. (1992)
15	2,7-dihydroxy-3,4,6-trimethoxyphenanthrene	*D. amplum*	Majumder et al. (2008)
		*D. rotundatum*	Majumder et al. (1992)
16	loddigesiinol A 7,10-dihydroxy-4,5-dimethoxyphenanthrene	*D. loddigesii*	Ito et al. (2010)
		^a^ *D. Sonia*	Cai (2020) ^b^
		*D. wardianum*	Zhang et al. (2017)
17	5-hydroxy-2,4-dimethoxyphenanthrene	*D. loddigesii*	Ito et al. (2010)
18	Plicatol A; 2,5-dihydroxy-4,9,10-trimethoxyphenanthrene	*D. nobile*	Yang et al. (2007)
		*D. plicatile*	Honda and Yamaki (2000)
19	4-methoxy-2,5,9-trihydroxyphenanthrene	*D. denneanum*	Lin et al. (2013)
20	2-methoxy-4,5-dihydroxyphenanthrene		
21	2,7-dihydroxyphenanthrene		
22	2,3,5-trihydroxy-4-methoxyphenanthrene	*D. thyrsiflorum*	Li et al. (2016)
23	2,7-dihydroxy-4-methoxyphenanthrene		
24	Fimbriol-A (2,5-dihydroxy-3,4,9-trimethoxyphenanthrene)		
25	3,4,7-trihydroxy-2-methoxyphenanthrene	*D. hainanense*	Zhang et al. (2015)
		*D. terminale*	Cheng et al. (2023)
26	2,8-dihydroxy-3,4,7-trimethoxyphenanthrene	*D. nobile*	Yang et al. (2007)
27	2,3,5-trihydroxy-4,9-dimethoxyphenanthrene		
28	2,5- dihydroxy-3,4-dimethoxyphenanthrene		
29	3-hydroxy-2,4,7-trimethoxyphenanthrene		
30	Bulbophyllanthrin (2-hydroxy-3,4,7-trimethoxyphenanthrene)		
31	dendroscabrol A; 1,5-dimethoxyphenanthrene-2,7-diol	*D. kingianum*	Takamiya et al. (2019)
		*D. scabrilingue*	Sarakulwattana et al. (2020)
		*D. speciosum*	Takamiya et al. (2019)
32	dendrocrumenol A 3,4,7-trihydroxy-2,8-dimethoxyphenanthrene	*D.crumenatum*	Kongkatitham et al. (2023)
33	2,7-dihydroxy-3,4,8-trimethoxyphenanthrene	*D. terminale*	Cheng et al. (2023)
34	4,7-dihydroxy-2,3-dimethoxyphenanthrene		
35	3,4,8-trimethoxyphenanthrene-2,5-diol	*D. nobile*	Hwang et al. (2010)
36	Fimbriol-B 2,3,5,9-tetrahydroxy-4-methoxyphenanthrene		
37	5,7-dimethoxyphenanthrene-2,6-diol		
38	dehydroorchinol (2,4-dimethoxy-7-hydroxyphenanthrene)	*D. nobile*	Kim et al. (2015)
39	1,5,7-dimethoxyphenanthren-2-ol		
40	1-methoxy-2,8-dihydroxyphenanthrene	*D.chrysotoxum*	Qian et al. (2025)
41	1,2,4,8-tetramethoxy-7- hydroxyphenanthrene		

^a^ horticultural cultivar (unlisted in POWO).

^b^ Chinese doctoral dissertation (unpublished). Other species have been listed in *Plants of the World Online* (POWO; http://www.plantsoftheworldonline.org).

^c^ Botanical variety.

^d^ Unlisted in POWO.

Chemotaxonomic patterns emerge from species-level distribution analysis (Table 1). Moscatin (6) exhibits the broadest distribution, reported in 24 species, followed by 2,5-dihydroxy-4,9-dimethoxyphenanthrene (11) in 10 species. Conversely, 22 compounds are single-species endemics, including 10 compounds specific in *D. nobile,* -and chrysotoxene (25) (*D. chrysotoxum*-specific), supporting its utility as species-diagnostic markers—an application direction not fully explored in previous reviews (Anuradha and Rao, 2022; Tóth et al., 2018).

### 2.2 Simple dihydrophenanthrenes

**FIGURE 3:**
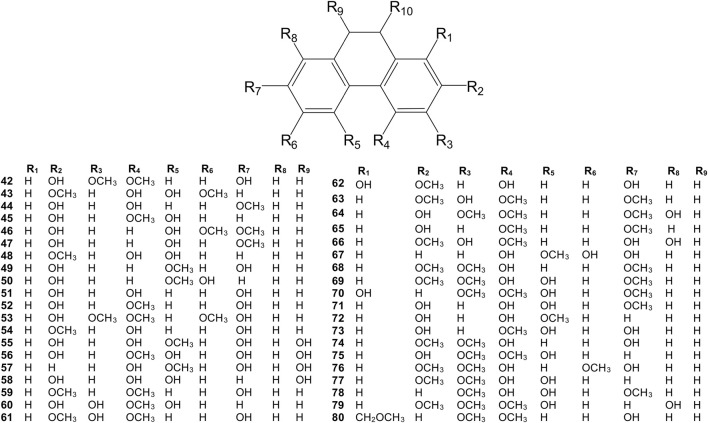
Chemical structures of simple dihydrophenanthrenes (42–80) isolated from the *Dendrobium* plants.

Dihydrophenanthrenes are the saturated counterparts of phenanthrenes, originating from formal hydrogenation of the C9–C10 double bond. This minor structural change abolishes central aromaticity, generating a 9,10-dihydro scaffold that retains the planar biphenyl upper moiety while introducing a flexible, non-aromatic ring (Tóth et al., 2018). Quantitative analysis of 41 simple 9,10-dihydrophenanthrenes (42–80) from 35 *Dendrobium* species reveals distinct substitution patterns compared to their phenanthrene counterparts (Figure 3; Table 2). Substitution shows that C-2, C-4, and C-7 position are dominantly oxygenated (hydroxylation: 71%, 20/39; 51%, 18/39; 46%, 46/39, respectively; methoxylation: 43%, 17/39 at C-4), with C-3 methoxylation at 30% (12/39). C-1, C-6, C-8, and C-9 remain sporadically oxygenated (<10% each). Notably, C-1 substitution by hydroxy was observed in compounds 62 and 70, a C-10 substituent has not been observed so far. The only alkyl modification is the ethyl-methyl substitution in compound 80. Compounds 51, and 58 are the sole examples bearing three or four hydroxy groups, and no exclusively methoxylated derivative has been reported to date.

**TABLE 2:** Thirty-nine simple dihydrophenanthrenes (42–80) and their distribution in *Dendrobium* plants.

No.	Compound name	Origin	References
42	erianthridin (3,4-dimethoxy-2,7-dihydroxy-9,10-dihydrophenanthrene)	*D. chrysotoxum*	Yang et al. (2001)
			Liu et al. (2022)
		*D. hainanense*	Zhang et al. (2015)
		*D. nobile*	Yang et al. (2007), Hwang et al. (2010), Zhou et al. (2016)
		*D. officinale*	Cui et al. (2019)
		*D. plicatile*	Yamaki and Honda (1996)
		^a^ *D. Sonia*	Cai (2020) ^b^
43	4,5-dihydroxy-2,6-dimethoxy-9,10-dihydrophenanthrene	*D. chrysotoxum*	Hu et al. (2012)
		*D. devonianum*	Wu et al. (2019)
44	2,4-dihydroxy-7-methoxy-9,10-dihydrophenanthrene	*D. aphyllum*	Yang et al. (2017)
		*D. chrysotoxum*	Liu et al. (2022)
45	hircinol (2,5-dihydroxy-4-methoxy-9,10-dihydrophenanthrene; 4-methoxy-9,10-dihydro-phenanthrene-2,5-diol; 9,10-dihydro-4-methoxy-2,5-phenanthrenediol)	*D. aphyllum*	Zhang et al. (2008)
		*D. chrysotoxum*	Liu et al. (2022)
		*D. chrysanthum*	Cai et al. (2017)
		*D. devonianum*	Meng et al. (2013)
		*D. draconis*	Sritularak et al. (2011)
		*D. fimbriatum*	Xu et al. (2014)
		*D. loddigesii*	Ito et al. (2010)
		*D. moniliforme*	Zhao et al. (2016)
		*D. moschatum*	Yang et al. (2022)
		*D. nobile*	Hwang et al. (2010)
			Zhou et al. (2016)
		*D. officinale*	Xu et al. (2022)
		*D. plicatile*	Chen et al. (2020)
		^a^ *D. Sonia*	Cai (2020) ^b^
		*D. thyrsiflorum*	Zhang et al. (2004); Zhang et al. (2005); Li et al. (2016)
46	ephemeranthol A (2,5-dihydroxy-6,7-dimethoxy-9,10-dihydrophenanthrene)	*D. gibsonii*	Thant et al. (2020)
		*D. infundibulum*	Ranong et al. (2019)
		*D. nobile*	Yang et al. (2007), Hwang et al. (2010), Kim et al. (2015), Zhou et al. (2016), Xu et al. (2018)
		*D. officinale*	Zhao et al. (2018), Cui et al. (2019)
		*D. plicatile*	Chen et al. (2020)
		*D. aphyllum*	Zhao et al. (2024)
47	lusianthridin (2,5-dihydroxy-7-methoxy-9,10-dihydrophenanthren)	*D. aphyllum*	Chen et al. (2008), Zhao et al. (2024)
		*D. brymerianum*	Klongkumnuankarn et al. (2015)
		*D. gibsonii*	Thant et al. (2020)
		*D. lindleyi*	Qin et al. (2022)
		*D. loddigesii*	Ito et al. (2010)
		*D. nobile*	Yang et al. (2007), Hwang et al. (2010), Zhou et al. (2016)
		*D. palpebrae*	Kyokong et al. (2019)
		*D. plicatile*	Chen et al. (2020)
		*D. scabrilingue*	Sarakulwattana et al. (2020)
		^a^ *D. Sonia*	Cai (2020) ^b^
		*D.venustum*	Sukphana et al. (2014)
48	Orchinol (4,5-dihydroxy-2-methoxy-9,10-dihydrophenanthrene)	^c^ *D. aurantiacum* Rchb. f. var. *denneanum*	Xu et al. (2016)
		*D. denneanum*	Wu et al. (2019)
		*D. nobile*	Hwang et al. (2010), Zhou et al. (2016)
		*D. officinale*	Zhao et al. (2018)
		^a^ *D. Sonia*	Cai (2020) ^b^
49	coelonin (2,7-dihydroxy-5-methoxy-9,10-dihydrophenanthrene)	*D. amplum*	Majumder et al. (2008)
		*D. aphyllum*	Chen et al. (2008)
		*D. heterocarpum*	Yang et al. (2019)
		*D. lindleyi*	Qin et al. (2022)
		*D. nobile*	Hwang et al. (2010), Yang et al. (2007)
		*D. plicatile*	Chen et al. (2020)
		*D. scabrilingue*	Sarakulwattana et al. (2020)
50	Flavanthridin (2,6-dihydroxy-5-methoxy-9,10-dihydrophenanthrene)	*D. nobile*	Xu et al. (2018)
		^a^ *D. Sonia*	Cai (2020) ^b^
51	2,4,7-trihydroxy-9,10-dihydrophenanthrenen (9,10-dihydrophenanthrene-2,4,7-triol)	*D. aphyllum*	Zhang et al. (2008)
		*D. fimbriatum*	Xu et al. (2014)
		*D. loddigesii*	Li et al. (2013)
		*D. officinale*	Zhao et al. (2018)
		*D. primulinum*	Ye et al. (2016)
		*D. polyanthum*	Hu et al. (2009)
52	2,7-dihydroxy-4-methoxy-9,10-dihydrophenanthrene (4-methoxy-9,10-dihydrophilic-2,7-diol)	*D. chrysotoxum*	Majumder and Sen (1987b)
		*D. wardianum*	Li et al. (2020)
53	2,7-dihydroxy-3,4,6-trimethoxy-9,10-dihydrophenanthrene	*D. rotundatum*	Majumder et al. (1992)
		*D. sinense*	Tan et al. (2017)
54	4,7-dihydroxy-2-methoxy-9,10-dihydroxyphenanthrene (2-methoxy-9,10-dihydrophenanthrene-4,7-diol)	*D. aphyllum*	Yang et al. (2017)
		*D. densiflorum*	Fan et al. (2001)
		*D. fimbriatum*	Xu et al. (2014)
		*D. nobile*	Lee et al. (1995)
55	5-methoxy-2,4,7,9S-tetrahydroxy-9,10-dihydrophenanthrene	*D. denneanum*	Lin et al. (2013)
56	4-methoxy-2,5,7,9S-tetrahydroxy-9,10-dihydrophenanthrene	*D. aphyllum*	Yang et al. (2017)
		*D. denneanum*	Lin et al. (2013)
57	5-methoxy-4,7,9S-trihydroxy-9,10-dihydrophenanthrene 5-methoxy-4,7,9-trihydroxy-9,10-dihydrophenanthrene	*D. aphyllum*	Yang et al. (2017)
		*D. denneanum*	Lin et al. (2013)
		^a^ *D. Sonia*	Cai (2020) ^b^
58	(s)-2,4,5,9-tetrahydroxy-9,10-dihydrophenanthrene	*D. fimbriatum*	Xu et al. (2014)
59	2,4-dimethoxy-9,10-dihydrophenanthren-7-ol		
60	2,3,5-trihydroxy-4-methoxy-9,10-dihydrophenanthrene	*D. thysiflorum*	Li et al. (2016)
61	3,7-dihydroxy-2,4-dimethoxy-9,10-dihydrophenanthrene	*D. plicatile*	Chen et al. (2020)
62	1,4,7-trihydroxy-2-methoxy-9,10-dihydrophenanthrene	*D. plicatile*	Chen et al. (2020)
		^a^ *D. Sonia*	Cai (2020) ^b^
63	3-hydroxy-2,4,7-trimethoxy-9,10-dihydrophenanthrene	*D. hainanense*	Zhang et al. (2015)
		*D. nobile*	Yang et al. (2007)
64	2,8-dihydroxy-3,4,7-trimethoxy-9,10-dihydrophenanthrene	*D. nobile*	Yang et al. (2007)
65	2-hydroxy- 4,7-dimethoxy-9,10-dihydrophenanthrene		
66	dendrocrumenol B (3,7,8-trihydroxy-2,4-methoxydihydrophenanthrene)	*D.crumenatum*	Kongkatitham et al. (2023)
67	epheneranthol C (4,6,7-trihydroxy-5-methoxy-9,10-dihydrophenanthrene)	*D. nobile*	Hwang et al. (2010)
68	dendroinfundin A (4-hydroxy-2,3,7-trimethoxy-9,10-dihydrophenanthrene)	*D. infundibulum*	Ranong et al. (2019)
69	dendroinfundin B (4,5-dihydroxy-2,3,8-trimethoxy-9,10-dihydrophenanthrene)	*D. infundibulum*	Ranong et al. (2019)
70	1,5-dihydroxy-3,4,7-trimethoxy-9,10-dihydrophenanthrene	*D. moniliforme*	Zhao et al. (2016)
71	7-methoxy-9,10-dihydrophenanthrene-2,4,5-triol	*D. draconis*	Sritularak et al. (2011)
		*D. longicornu*	Hu et al. (2008)
72	2,4-dihydroxy-5-methoxy-9,10-dihydrophenanthrene	*D. chrysanthum*	Cai et al. (2017)
73	2,5,7-trihydroxy-4-methoxy-9,10-dihydrophenanthrene	*D. bellatulum*	Shang et al. (2019)
		*D. sinense*	Tan et al. (2017)
74	4,7-dihydroxy-2,3-dimethoxy-9,10-dihydrophenanthrene	*D. sinense*	Chen et al. (2013)
			Tan et al. (2017)
		^a^ *D. Sonia*	Cai (2020) ^b^
75	2,5-dihydroxy-3,4-trimethoxy-9,10-dihydrophenanthrene	*D. sinense*	Tan et al. (2017)
76	4,7-dihydroxy-2,3,6-trimethoxy-9,10-dihydrophenanthrene	*D. sinense*	Chen et al. (2013)
77	4,5-dihydroxy-2,3-dimethoxy-9,10-dihydro-phenanthrene		
		*D. pachyglossum*	Warinhomhoun et al. (2021)
78	4,5-dihydroxy-3,7-dimethoxy-9,10-dihydrophenanthrene	*D. ellipsophyllum*	Kasinee et al. (2014)
		*D. nobile*	Ye and Zhao (2002)
		*D. wardianum*	Li et al. (2024)
79	calanhydroquinone C 5,8-dihydroxy-2,3,4-trimethoxy-9,10-dihydrophenanthrene)	*D. plicatile*	Chen et al. (2020)
80	3,4-dimethoxy-1-(methoxymethyl)-9,10-dihydrophenanthrene-2,7-dio	*D. hainanense*	Zhang et al. (2019)

^a^ Horticultural cultivar (unlisted in POWO).

^b^ Chinese doctoral dissertation (unpublished). Other species have been listed in *Plants of the World Online* (POWO; http://www.plantsoftheworldonline.org).

^c^ Botanical variety (Synonym of *D. aurantiacum* var. *denneanum*;

Chemotaxonomic patterns reflect biosynthetic accessibility. Hircinol (45) is the most widely distributed metabolite, reported in 14 species, followed by ephemeranthol A (46) (14 species), lusianthreidin (47) (11 species), coelonin (49) (7 species) and 2,4,7-trihydroxy-9,10-dihydrophenanthrene (51) (5 species). Conversely, 18 compounds are single-species endemics, including dendrocrumenol B (66) (*D. crumenatum*-specific) and epheneranthol C (67) (*D. noble-*specific), expanding species-diagnostic marker repertoire beyond phenanthrene profiles (Table 2).

### 2.3 Diphenanthrenes

Diphenanthrenes constitute a rare but structurally intriguing class of C–C-linked dimers in which two phenanthrene or dihydrophenanthrene units are joined, typically through their C-2/C-2′, C-4/C-4′ or C-7/C-7′ positions (Yao et al., 2008; Tóth et al., 2018). These linkages generate a rigid, bi-axially chiral scaffold that extends the π-conjugation system while preserving the oxygenation patterns commonly observed in monomeric precursors. To date, only three phenanthrene homodimers (81–83) have been identified from the genus, nine dihydrophenanthrene homodimers (84–92) and three phenanthrene-dihydrophenanthrene heterdimers (93–95) have been characterized (Supplementary Figure S1; Table 3).

**TABLE 3:** Fifteen diphenanthrenes including three phenanthrene homodimers (81–83), nine dihydrophenanthrene homodimers (84–92), three phenanthrene-dihydrophenanthrene heterdimers (93–95) and their distribution in *Dendrobium* plants.

No.	Compound name	Origin	References
81	denthyrsinol; 4,5’ -dimethoxy-[1,10 ]biphenan threnyl-2,5,4’,7’ -tetraol	*D. nobile*	Zhou et al. (2016)
		*D. primulinum*	Ye et al. (2016)
		*D. thyrsiflorum*	Zhang et al. (2005)
82	Denchrysoin A (4,4′-dimethoxy-9,10-dihydro-[1,1′-biphenanthrene]-2,7,2′-triol)	*D. chrysotoxum*	Qian et al. (2025)
83	Denchrysoin B (4,4′,7-trimethoxy-9,10-dihydro-[1,2′-biphenanthrene]-2,7′-diol)	​	Zhang et al. (2005)
84	Phoyunnanin C	*D. venustum*	Sukphana et al. (2014)
85	Phoyunnanin E	​	
86	4,4′,7,7′-tetrahydroxy-2,2′-dimethoxy-9,9′,10,10′-tetrahydro-1,1′-phenanthrene	*D. nobile*	Zhou et al. (2016)
87	Phochinenin D		Yang et al. (2007)
88	Phochinenin G		
89	Denchrysoin D	*D. chrysotoxum*	Qian et al. (2025)
90	Flavanthrin	*D. aphyllum*	Chen et al. (2008)
91	2,2′-dimethoxy-3,3′,4,4′,7,7′-tetrahydroxy-9,9′,10,10′-tetrahydro-1,1′-biphenanthrene (95	*D. nobile*	Zhou et al. (2016)
			Yang et al. (2007)
92	Amplumthrin	*D. amplum*	Majumder et al. (2008)
93	denthyrsinol C	*D. nobile*	Zhou et al. (2016)
94	denthyrsinol A (2,4-dimethoxy-9,10-dihydrophenanthren-1-yl)-4-methoxyphenanthrene-2,7-diol)	*D. nobile*	Zhou et al. (2016)
95	denthyrsinol B	​	Yang et al. (2007)

All species have been listed in *Plants of the World Online* (POWO; http://www.plantsoftheworldonline.org).

### 2.4 Phenanthraquinones

Phenanthraquinone is the two-electron oxidation product of phenanthrene. Dehydrogenation at the 9,10-bond converts the central aromatic ring into an ortho-quinonoid diketone, forming a constitutional isomer series where phenanthraquinone, phenanthrene, and 9,10-dihydrophenanthrene differ only in the oxidation level at the pivotal C-9/C-10 bonds (Anuradha and Rao, 2022). The quinone retains the fully aromatic outer rings but replaces the 9,10-π-system with a conjugated 1,2-dicarbonyl, creating an electron-deficient center. Electron-donating substituents (OH, OMe, methylenedioxy) at C-2, C-3, C-6, and C-7 donate electron density through resonance (+M effect), partially compensating for the electron-withdrawing nature of the 1,2-dicarbonyl and modulating redox potential. Additional hydroxylation or glycosylation at C-4 or C-7 further diversifies the structure (Guedouar et al., 2017).

**FIGURE 4:**
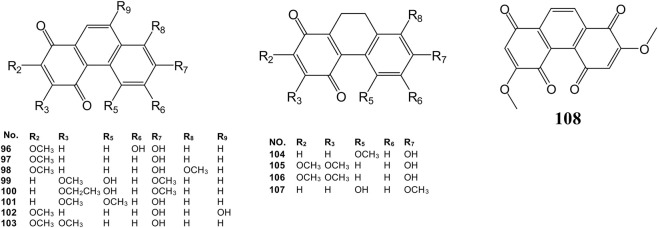
Chemical structures of phenanthraquinone (96–108) isolated from the *Dendrobium* plants.

Seventeen members (96–113) have been isolated from *Dendrobium* species (Figure 4; Table 4). Compounds 96–103 are 1,4-phenanthraquinones, while compounds 104–107 represent a 9,10-dihydro-1,4-phenanthraquinone (Figure 4; Table 4). Moniliformin (108) is the sole 1,4,5,8-phenanthradiquinone reported so far, which is isolated specifically from *D. moniliforme*. Compounds 109–113 (Supplementary Figure S1; Table 4) constitute phenanthraquinone dimers, a rare but structurally distinct subset of orchid-derived phenanthrenoids. These dimers form through C–C or C–O–C coupling of two phenanthraquinone units (or a phenanthraquinone with a phenanthrene), most commonly at C-2, C-3, C-4, or C-7 positions. In *Dendrobium* species, denthyrsinone (109) and dendropalpebrone (110) are 1,4-phenanthraquinone-phenanthrene dimers from *D. thyrsiflorum* and *D. palpebrae*, respectively. Loddigesiinol G (111) and H (112), and dendrocandin H (113) are 1,4-phenanthraquinone-bibenzyl hereodimers from *D. loddigesii* and *D. candidum*. Thus, the conversion of the 9,10-bond into an ortho-quinonoid diketone not only extends the π-conjugation to produce the characteristic orange-red chromophore and reversible redox chemistry, but also establishes a unique structural platform for further regioselective functionalization, positioning phenanthraquinones as the most oxidized and chromogenically distinct members within the *Dendrobium* phenanthrene family.

**TABLE 4:** Seventeen phenanthraquinones including thirteen phenanthraquinones (96–108), four phenanthraquinone dimers (109–113) and their distribution in *Dendrobium* plants.

No.	Compound name	Origin	References
96	6,7-dihydroxy-2-methoxy-1,4-phenanthrenedione	*D. nobile*	Zhou et al. (2016)
97	densiflorol B (ephemeranthoquinone; 7-hydroxy-2-methoxy-1,4-phenanthrenedione)	*D. nobile*	Zhou et al. (2016), Zhou et al. (2018)
		^a^ *D. Sonia*	(Cai, 2020)*
		*D. chrysotoxum*	Li et al. (2009)
		*D. densiflorum*	Fan et al. (2000), Fan et al. (2001)
		^c^ *D. hongdie*	Chen et al. (2015)
		*D. plicatile*	Yamaki and Honda (1996)
		*D. venustum*	Sukphana et al. (2014)
		*D. lindleyi*	Qin et al. (2022)
		*D. crumenatum*	Kongkatitham et al. (2023)
		*D. thyrsiflorum*	Liu et al. (2011)
		*D. brymerianum*	Chen et al. (2014)
98	cypripedin (7-hydroxy-2,8-dimethoxy-1,4-phenanthrenedione)	*D. nobile*	Zhou et al. (2018)
		*D. densiflorum*	Fan et al. (2001)
		*D. lindleyi*	Qin et al. (2022)
		*D. crumenatum*	Kongkatitham et al. (2023)
99	Denbinobin (5-hydroxy-3,7-dimethoxy-1,4-phenanthraquinone)	*D. wardianum*	Zhang et al. (2017)
			Li et al. (2020)
		*D. nobile*	Yang et al. (2007)
			Lee et al. (1995)
		*D. thyrsiflorum*	Liu et al. (2011)
		*D. officinale*	Xu et al. (2022)
		*D. moniliforme*	Lin et al. (2001), Bae et al. (2004)
100	3-ethoxy-5-hydroxy-7-methoxy-1,4-phenanthraquinone	*D. wardianum*	Li et al. (2020)
101	7-hydroxy-5,6-dimethoxy-1,4-phenanthrenequinone	*D. moniliforme*	Bae et al. (2004)
102	dengraphenol N	*D.gratiosissimum*	Li et al. (2022)
103	2,3-dimethoxyl-7-hydroxyl-1,4-phenanthrenedione	*D. terminale*	Cheng et al. (2023)
		^a^ *D. Sonia*	(Cai, 2020)*
104	5- methoxy-7-hydroxy-9,10-dihydro-1,4-phenanthrenequinone (9,10-dihydro-7-hydroxy-5-methoxy-1,4-phenanthrenedione)	*D. draconis*	Hu et al. (2008)
		*D. officinale*	Xu et al. (2022)
105	Denbinobin B (9,10-dihydro-7-hydroxy-2,3-dimethoxy-1,4-phenanthrenedione)	^a^ *D. Sonia*	(Cai, 2020)*
		*D. officinale*	Xu et al. (2022)
		*D. sinense*	Chen et al. (2013)
106	5-hydroxy-7-methoxy-9,10-dihydrophenanthrene-1,4-dione	*D. thyrsiflorum*	Liu et al. (2011)
		*D. longicornu*	Hu et al. (2008)
107	dedronone (5-hydroxyl-7-methoxy-9,10-dihydro-1,4-phenanthrenequinone)	*D. cariniferum*	Chen et al. (2008)
108	moniliformin/moniliformediquinone (2,6-dimethoxy-1,4,5,8-phenanthradiquinone)	*D. moniliforme*	Lin et al. (2001)
109	denthyrsinone (7,4′,7′-trihydroxy-2,2′,8′-trimethoxy-[5,1´]-biphenanthrenyl-1,4-dione)	*D. thyrsiflorum*	Zhang et al. (2005)
110	dendropalpebrone	*D. palpebrae*	Kyokong et al. (2019)
111	loddigesiinol G ((S)-3-[(1S)-1-(4-hydroxy-3-methoxyphenyl)-2-(4-hydroxy-3,5-dimethoxyphenyl)ethyl]-7-hydroxyphenanthrene-1,4-dione)	*D. loddigesii*	Lu et al. (2014)
112	loddigesiinol H	*D. loddigesii*	Lu et al. (2014)
113	dendrocandin H; (*S*)-5-[2-(3,4-dihydroxyphenyl)ethyl]-3,4-dimethoxybenzene-1,2-diol	^d^ *D. candidum*	Li et al. (2009)

a Horticultural cultivar (unlisted in POWO); c. unlisted in POWO; d, botanical variety (Synonym of *D. moniliforme*; *. Chinese doctoral dissertation (unpublished). Other species have been listed in *Plants of the World Online* (POWO; http://www.plantsoftheworldonline.org).

### 2.5 Phenanthrene glycosides

To date, ten phenanthrene glycosides (114–123) have been characterized across the genus (Supplementary Figure S1; Table 5). Among them, nine were isolated from *D. denneanum*, including five monoglycosides (114–118) and four diglycosides (120–123). A single diglycoside (119) was obtained from *D. chrysanthum*. Only three dihydrophenanthrene glycosides (124–126) have been reported in three *Dendrobium* species so far (Supplementary Figure S2; Table 4). All these compounds share a β-D-glucopyranosyl moiety attached to C-2, C-4 or C-5, suggesting regioselective glycosylation during biosynthesis. Hence, the regioselective installation of a β-D-glucopyranosyl unit on the phenanthrene or 9,10-dihydrophenanthrene core not only increases molecular polarity and aqueous solubility but also masks a key phenolic pharmacophore, offering a tunable balance between membrane permeability and target-site recognition that underlies the distinctive bioactivity profile of this rare glycosylated subclass.

**TABLE 5:** Thirteen phenanthrene glycosides (114–126) and their distribution in *Dendrobium* plants.

No.	Compound name	Origin	References
114	2,5-dihydroxy-4-methoxy-phenanthrene2-*O-β-*_*D*_-glucopyranoside	*D. denneanum*	Lin et al. (2013)
115	2,5-dihydroxy-4-methoxy-phenanthrene 2-*O*-*β-*_*D*_-apiofuranosyl-(1–6)-*β-*_*D*_-glucopyranoside		
116	2,5-dihydroxy-4-methoxy-phenanthrene 2-*O*-α-_L_-rhamnopyranosyl-(1–6)-β*-*_*D*_-glucopyranoside		
117	denneanoside A (2,5-dihydroxy-4-methoxyphenanthrene 2-*O*-β-_D_-glucopyranoside)	*D. denneanum*	Fu et al. (2013)
118	denneanoside F 2,5,9-trihydroxy-4-methoxy-9,10-didydrophenanthrene-2-*O-β-D*-glucopyranoside		
119	denchryside A (2,6-dihydroxy-1,5,7-trimethoxyphenanthrene-2-*O*-[*α-L*-rhamnopyranosyl-1→6]-β-_D_-glucopyranoside)	*D. chrysanthum*	Ye et al. (2003)
120	denneanoside B (2,5-dihydroxy-4-methoxyphenanthrene-2-*O-*β*-*_D_-glucopyranosyl-(1→6)-β*-*_D_-glucopyranoside)	*D. denneanum*	Fu et al. (2013)
121	denneanoside C (2,5-dihydroxy-4-methoxyphenanthrene-2-*O-*β*-*_D_-glucopyranosyl-(1→2)-β-_D_-glucopyranoside)		
122	denneanoside D (2,5-dihydroxy-4-methoxyphenanthrene-2-*O-*α-_L_-rhamnopyranosyl-(1→6)-β-_D_-glucopyranoside)		
123	denneanoside E [2,5-dihydroxy-4-methoxyphenanthrene-2-*O*-β-_D_-apiofuranosyl-(1→6)-β-_D_-glucopyranoside]		
124	1,2,5,9R-tetrahydroxy-9,10-dihydrophenanthrene-5-*O-β-D*-glucopyranoside	*D. denneanum*	Lin et al. (2013)
		*D. nobile*	Zhou et al. (2017)
125	4-methoxy-2,5,9R-trihydroxy-9,10-dihydrophenanthrene 2-*O-β-D*-glucopyranoside	*D. denneanum*	Lin et al. (2013)
126	2,4,5,9S-tetrahydroxy-9,10-dihydrophenanthrene 4-*O-β-D*-glucopyranoside	*D. primulinum*	Ye et al. (2016)

All species have been listed in *Plants of the World Online* (POWO; http://www.plantsoftheworldonline.org).

### 2.6 Phenanthrene heterodimers

Phenanthrene heterodimers are asymmetric dimers produced by C-C or C-O-C cross-coupling of a phenanthrene or 9,10-dihydrophenanthrene unit with a structurally different aromatic or heterocyclic fragment, such as fluorene, tetrahydropyran, dihydrofuran, or a formaldehyde-derived pyrrole. The resulting scaffolds display bridged, spiro-fused, or condensed topologies that impose marked steric hindrance and modulate electronic properties (Yao et al., 2008). Representative phenanthrene heterodimers include loddigesiinol I (127) and B (128) obtained from *D. loddigesii*, and denchrysoin F (129) obtained from *D. chrysotoxum*. The phenanthrene-formaldopyrrole dendrocandin P1 (130) was exclusively identified in *D. officinale* (Zhao et al., 2018) (Supplementary Figure S2; Table 6).

**TABLE 6:** Four phenanthrene heterodimers (127–130), ten dihydrophenanthrene heterodimers (131–140), eight spiro-phenanthrenes (141–148) and their distribution in *Dendrobium* plants.

No.	Compound name	Origin	References
127	loddigesiinol I	*D. loddigesii*	Ito et al. (2010)
128	loddigesiinol B		Lu et al. (2014)
129	Denchrysoin F	*D. chrysotoxum*	Qian et al. (2025)
130	dendrocandin P1; (2*S*, 3*S*)-2-(-4-hydroxy-3 5-dimethoxyphenyl)-3-hydroxymethyl-2, 3-dihydrophenanthro [1, 4] dioxin-8-ol	*D. officinale*	Zhao et al. (2018)
131	loddigesiinol J	*D. loddigesii*	Lu et al. (2014)
		*D. nobile*	Zhou et al. (2017)
132	(2R*,3S*)-3-hydroxymethyl-9-methoxy-2-(4′-hydroxy-3′,5′-dimethoxyphenyl)-2,3,6,7-tetrahydrophenanthro- [4,3-b]furan-5,11-diol	*D. moniliforme*	Zhao et al. (2016)
133	dendronbibisline A	*D. nobile*	Cheng et al. (2020)
134	dendronbibisline B		
135	Denchrysoin E	*D. chrysotoxum*	Hu et al. (2012)
136	chrysotoxol A; *trans*-2-(4-hydroxy-3-methoxyphenyl)-2,3,5,6-tetrahydrophenanthro [2,1-*b*]pyran-3,8,11-triol		Qian et al. (2025)
137	chrysotoxol B; (12*S*,13*R*)-2-(4-hydroxy-3,5-dimethoxyphenyl)-2,3,5,6-tetrahydrophenanthro [2,1-*b*]pyran-3,8,11-triol		
138	dendrosignatol	*D. signatum*	Mittraphaba et al. (2016)
139	dendrogibsol	*D. gibsonii*	Thant et al. (2020)
140	dendrocandin P2; (2*S*, 3*S*)-2-(-4-hydroxy-3,5-dimethoxyphenyl)-3-hydroxymethyl-2,3,10,11 -tetrahydro-phenanthro [1, 4] dioxin-8-ol	*D. officinale*	Zhao et al. (2018)
		^a^ *D. Sonia*	Cai (2020) ^b^
141	dendrochrysanene A	*D. chrysotoxum*	Qian et al. (2025)
142	dendrochrysanene B		
143	dendrochrysanene C		
144	dendrochrysanene D		
145	dendrochrysanene E		
146	dendrochrysanene F		
147	dendrochrysanene G		
148	Dendrochrysanene; 2-hydro-7,7′,10′-trihydroxy-4-4′-dimethoxylspiro [(1*H*)-cyclopenta [a]naphthalene-3,3’- (20*H*)-phenanthro [2,1-*b*]furan]-1,2′-dione	*D. chrysanthum*	Yang et al. (2006)

a Horticultural cultivar (unlisted in POWO).

^b^ Chinese doctoral dissertation (unpublished). Other species have been listed in *Plants of the World Online* (POWO; http://www.plantsoftheworldonline.org).

Ten dihydrophenanthrene-based heterodimers (131–140) fusing a 9,10-dihydrophenanthrene to a heterocyclic or carbocyclic unit have been identified to date (Supplementary Figure S2; Table 6). Four bear a dihydrofuran ring, namely loddigesiinol J (131) from *D. loddigesii* (Lu et al., 2014), the tetrahydrofuran 132 from *D. moniliforme* (Zhao et al., 2016), and dendronbibisline A (133) and B (134) from *D. nobile* (Cheng et al., 2020). Denchrysoin E (135), chrysotoxol A (136) and B (137) from *D. chrysotoxum* (Hu et al., 2012; Qian et al., 2025), dendrosignatol (138) from *D. signatum* (Mittraphaba et al., 2016). The phenanthrene-fluorene polymer dendrogibsol (139) was identified in *D. gibsonii* (Thant et al., 2020)*,* and dendrocandin P2 (140), each bearing an oxygen heterocycle, were derived from *D. Sonia* (Cai, 2020) and *D. officinale* (Zhao et al., 2018), respectively. These structures underscore the biosynthetic capacity of the genus to generate diverse heterodimeric scaffolds with potential pharmacological value. These hybrid scaffolds underscore the biosynthetic versatility of *Dendrobium* and provide a rich structural platform for exploring novel bioactivities.

### 2.7 Spiro-phenanthrenes

Spiro-phenanthrenes feature a cyclopentane ring fused to the phenanthrene nucleus through a single sp^3^ quaternary carbon. This arrangement locks the molecule into a rigid, non-planar conformation and introduces axial chirality, while the spiro junction interrupts the extended π-system and generates distinctive stereoelectronic properties (Qian et al., 2025). To date, sevenof such compounds have been reported, including dendrochrysanene A-G (141–147) derived from *D. chrysotoxum* (Qian et al., 2025) and dendrochrysanene (148) derived from *D. chrysanthum* (Yang et al., 2006) (Supplementary Figure S2; Table 6). They differ only in terms of the hydroxy/methoxy substitution pattern around the spiro center. Collectively, these findings establish spiro-phenanthrenes as a scarce yet architecturally unique class within the genus, whose rigid, chiral spiro-centre not only breaks conjugation but also offers a novel stereochemical template for future expansion and synthetic exploitation.

### 2.8 Phenanthrene derivatives

Phenanthrene lactones (149–153) and phenanthrenone-type analogues (154–158) are phenanthrene derivatives isolated from plants of the genus *Dendrobium* (Supplementary Figure S2; Table 7). Phenanthrene-fused lactones are natural product hybrids combining a phenanthrene scaffold with a lactone functionality, coupled with the metabolic stability conferred by the lactone ring, may contribute to improve membrane permeability and oral bioavailability (Koyama et al., 2025; Tóth et al., 2018). Only five representatives have been identified from the genus so far. Specifically, fimbriatone (149) was isolated from *D. chrysotoxum, D. fimbriatum* and *D. nobile*, and crystalltone (150) was isolated from *D. crystallinum*. Lavidin (151) was obtained from *D. pierardii*, whereas imbricatin (152) and flaccidin (153) were isolated from *D. amoenum*. 152 was also detected in *D. herbaceum*.

**TABLE 7:** Six phenanthrene lactones (149–154), four phenanthrenone-type analogues (155–158) and their distribution in *Dendrobium* plants.

No.	Compound name	Origin	References
149	Fimbriatone	*D. chrysotoxum*	Liu et al. (2022)
		*D. fimbriatum*	Bi et al. (2003)
		*D. nobile*	Zhang et al. (2008)
150	crystalltone; 2-ethoxy-1-hydroxy-7-methoxy-5*H*-naphtho [8,1,2-cde]chromen-5- one	*D. crystallinum*	Wang et al. (2009)
151	lavidin	*D. pierardii*	Veerraju et al. (1955)
152	Imbricatin	*D. amoenum*	
		*D. herbaceum*	
153	Flaccidin	*D. amoenum*	
154	dendrodevonin A; (1*R*)-5-dihydroxyl-7-methoxy-4-one	*D. devonianum*	Wu et al. (2019)
155	dendrodevonin B; (1*R*)-5-dihydroxyl-7-methoxy-9,10-dihydrophenanthrene-4-one		
156	denobilone B; (4a*R*,10a*R*)-4a,5-dihydroxy-4-methoxy-1,9,10,10a-tetahydro-2-(4a*H*)-phenanthreneone	*D. nobile*	Zhou et al. (2016)
157	denobilone C; (4a*R*,10a*S*)-4a,5-dihydroxy-4-methoxy-10,10a-dihydro-2,9-(1*H*,4a*H*)-phenanthrenedione		
158	Aphyllone A	*D. aphyllum*	Yang et al. (2015)

All species have been listed in *Plants of the World Online* (POWO; http://www.plantsoftheworldonline.org).

Phenanthrenones are simple (or 9,10-dihydro) phenanthrenes bearing a single aryl ketone, usually at C-4. They are produced by the oxidation of the corresponding phenol. The planar, conjugated ketone enhances radical-scavenging activity. Five phenanthrenone derivatives (154–158) have been identified in several *Dendrobium* species (Supplementary Figure S2; Table 7). Dendrodevonin A (154) and B (155) were isolated from *D. devonianum*, denobilone B (156) and denobilone C (157) were isolated from *D. nobile*, and aphyllone A (158) was isolated from *D. aphyllum*.

These derivatives exhibit non-canonical substitution patterns or ring modifications, further expanding the chemical space of *Dendrobium* phenanthrenes. Collectively, the scarce yet structurally sharp lactone and ketone branches, exemplified by only ten representatives across both subclasses, illustrate that *Dendrobium* can further sculpt the phenanthrene nucleus through oxidative ring formation or carbonyl insertion. This strategy expands its chemical repertoire beyond simple oxygenation and offers additional pharmacophoric handles for lead optimisation.

### 2.9 Biosynthetic implications

The eight structural classes of *Dendrobium* phenanthrenes derive from an oxidative hierarchy: bibenzyls → 9,10-dihydrophenanthrenes → phenanthrenes → phenanthraquinones (Anuradha and Rao, 2022). This pathway is initiated by bibenzyl synthase (BBS)-catalyzed oxidative coupling of phenylpropanoid units, followed by sequential dehydrogenase and oxidase catalysis. The co-occurrence of multiple oxidation states across 49 species suggests enzymatic promiscuity in this pathway. Beyond monomeric diversification, oxidative coupling of preassembled phenanthrene units at electron-rich positions (C-2, C-4, C-7) generates dimeric architectures, extending the structural repertoire to biphenanthrenes and heterodimers (Takamiya et al., 2019). Late-stage tailoring reactions, particularly glycosylation, exhibit differential distribution across oxidation classes—absent in simple phenanthrenes but prevalent in more oxidized forms—possibly reflecting substrate accessibility or compartmentalization effects. These insights provide a roadmap for synthetic biology approaches to engineer non-natural phenanthrene analogs.

## 3 Bioactivities

Recent pharmacological studies have revealed that phenanthrene compounds isolated from *Dendrobium s*pecies exhibit remarkable anti-tumor and anti-inflammatory activities. Accumulating evidence supports their therapeutic potential through antioxidant, hypoglycemic, lipid-lowering, anti-fibrotic, and antiplatelet activities, making them potential candidates for multifaceted pharmacological interventions. A five-tier classification system was established for the systematic evaluation of biological activities of *Dendrobium* phenanthrenes. This framework comprises: Level 1 (preliminary activity), based on a single cell line with IC_50_ < 100 μM; Level 2 (validated activity), based on ≥2 cell lines with IC_50_ < 50 μM and dose-dependent response; Level 3 (mechanism-based activity), confirmed by target engagement validation and signaling pathway modulation; Level 4 (*in vivo* activity), verified by animal models. This classification system enables systematic integration of quantitative data while fully accounting for inter-study heterogeneity. Among 158 reported phenanthrene metabolites, 64 compounds (64/158, 40.5%) have been evaluated in at least one bioactivity assay, with reported activities spanning anti-tumor (cytotoxic), anti-inflammatory, antioxidant, antidiabetic, anti-fibrotic, antiplatelet, or antimicrobial endpoints. 54 compounds (54/64, 84.4%) met at least one criterion, whereas only 4 compounds (4/64, 6.2%) achieved Level 4 status, with none reaching Level 5. This stratified presentation avoids misleading precision while providing actionable theoretical guidance for prioritizing compounds in subsequent development efforts (Supplementary Table S1).

### 3.1 Antioxidant and anti-inflammatory properties

Oxidative stress and inflammation constitute a self-amplifying pathological cycle that drives tissue injury and promotes the development and progression of metabolic disorders, atherosclerosis, neurodegeneration, and cancer (Altanam et al., 2025). Within the *Dendrobium* phenanthrene series, eleven derivatives exhibited moderate 1,1-diphenyl-2-picrylhydrazyl (DPPH) radical-scavenging activity (Table 8), while twenty-seven compounds effectively suppressed lipopolysaccharide (LPS)-induced production of nitric oxide (NO) in macrophages (Table 9). Among these anti-inflammatory agents, compound 124 exhibited the most potent activity with an IC_50_ of 0.7 µM (Lin et al., 2013). Nine additional analogues, including compounds 6, 16, 17 (Ito et al., 2010), 20, 47, 55, 56, 114 (Hwang et al., 2010; Ito et al., 2010; Lin et al., 2013), showed robust inhibitory effects (IC_50_ 2.6–7.6 µM), underscoring the substantial anti-inflammatory potential of this class of compounds.

**TABLE 8:** Antioxidant effects of eleven *Dendrobium* phenanthrenes through scavenging DPPH free radical.

No.	Compound (No.)	IC_50_ (*μ*M)	References
1	confusarin (2)	12.90 ± 0.35	Zhang et al. (2008)
2	epheranthol B (9)	29.67 ± 1.11	
3	2,5-dihydroxy-4,9-dimethoxyphenanthrene (11)	34.78 ± 0.04	
4	Flavanthrinin (12)	35.71 ± 0.19	
5	Fimbriatone (149)	144.50 ± 2.65	
6	moscatin (6)	59.8	Ito et al. (2010)
7	loddigesiinol A (16)	26.1	
8	lusianthridin (47)	62.2	
9	hircinol (45)	22.3 ± 1.0	Sritularak et al. (2011)
10	7-methoxy-9,10-dihydrophenanthrene-2,4,5-triol (71)	10.2 ± 0.1	
11	5-methoxy-7-hydroxy-9,10-dihydro-1,4-phenanthrenequinone (104)	283.3 ± 13.7	

**TABLE 9:** Anti-inflammatory effects of 26 *Dendrobium* phenanthrenes by inhibiting LPS-induced NO production in RAW264.7 macrophages.

No.	Compound (No.)	IC_50_ (*μ*M)	References
1	confusarin (2)	29.2 ± 2.6	Liu et al. (2022)
2	7-hydroxy-2,3,4-trimethoxyphenanthrene (3)	9.4 ± 0.9	
3	moscatin (6)	6.4	Ito et al. (2010)
		6.3	Lin et al. (2013)
4	Nudol (10)	21.1 ± 3.8	Liu et al. (2022)
5	Rotundatin (14)	29.1	Ito et al. (2010)
6	loddigesiinols A (16)	2.6	
7	5-hydroxy-2,4-dimethoxyphenanthrene (17)	5.3	
8	4-methoxy-2,5,9-trihydroxyphenanthrene (19)	27.4	Lin et al. (2013)
9	2-methoxy-4,5-dihydroxyphenanthrene (20)	7.6	
10	2,7-dihydroxyphenanthrene (21)	32.8	
11	3,4,8-trimethoxyphenanthrene-2,5-diol (35)	20.4 ± 0.8	Hwang et al. (2010)
12	Fimbriol-B (36)	28.9 ± 0.6	
13	5,7-dimethoxyphenanthrene-2,6-diol (37)	35.7 ± 0.6	
14	2,4-dihydroxy-7-methoxy-9,10-dihydrophenanthrene (44)	25.9 ± 4.5	Liu et al. (2022)
15	hircinol (45)	29.2;	Ito et al. (2010)
		26.4 ± 0.2	Hwang et al. (2010)
16	ephemeranthol A (46)	12.0 ± 0.3	Hwang et al. (2010)
17	lusianthridin (47)	4.6	Ito et al. (2010)
18	coelonin (49)	10.2 ± 0.2	Hwang et al. (2010)
19	Flavanthridin (50)	34.1 ± 0.9	
20	5-methoxy-2,4,7,9S-tetrahydroxy-9,10-dihydrophenanthrene (55)	3.1	Lin et al. (2013)
21	4-methoxy-2,5,7,9S-tetrahydroxy-9,10-dihydrophenanthrene (56)	4.2	
22	epheneranthol C (67)	17.6 ± 0.4	Hwang et al. (2010)
23	2,5-dihydroxy-4-methoxy-phenanthrene 2-*O-β-D*-glucopyranoside (114)	4.6	Lin et al. (2013)
24	2,5-dihydroxy-4-methoxy-phenanthrene 2-*O-β-D*-apiofuranosyl-(1–6)- *β-D*-glucopyranoside (115)	16.9	
25	2,5-dihydroxy-4-methoxy-phenanthrene2-*O-α-L*-rhamnopyranosyl-(1–6)*-β-D*- glucopyranoside (116)	41.5	
26	1,2,5,9R-tetrahydroxy-9,10-dihydrophenanthrene 5-*O-β-D*-glucopyranoside (124)	0.7	Lin et al. (2013)

**FIGURE 5:**
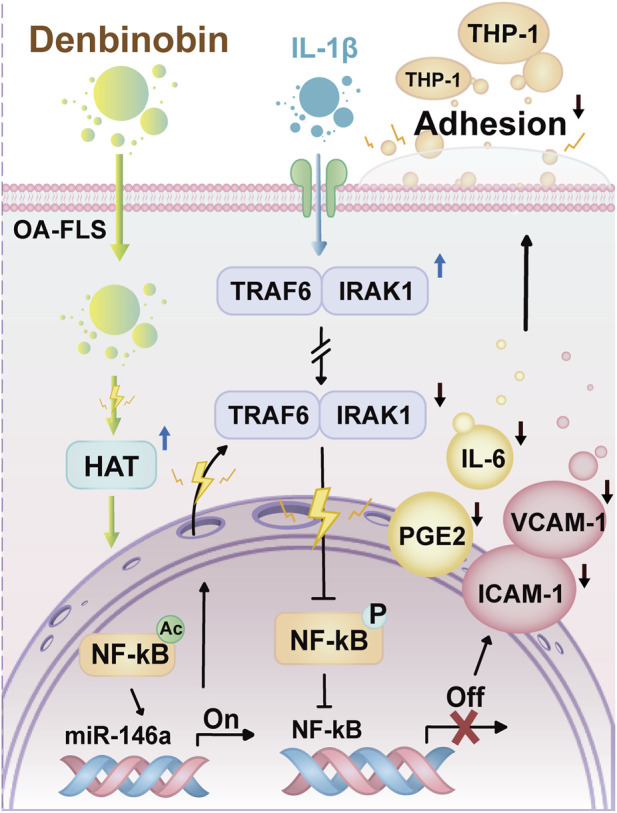
Denbinobin (147) upregulates miR-146a expression and attenuates IL-1β-induced inflammatory response.

Mechanistically, *Dendrobium* phenanthrenes suppressed LPS-induced inflammation by inhibiting the nuclear factor B (NF-κB) and mitogen-activated protein kinases (MAPK) [p38, jun N-terminal kinase (JNK), extracellular regulated protein kinases (ERK)] signaling axes (Table 10), thereby downregulating inducible nitric oxide synthase (iNOS)/cyclooxygenase-2 (COX-2) expression and the downstream release of nitric oxide (NO), prostaglandin E_2_ (PGE_2_), tumor necrosis factor (TNF-α), interleukin-1β (IL-1β), and (interleukin-6) IL-6. Notably, denbinobin (99) reinforced this anti-inflammatory effect through miR-146a-mediated repression of adhesion molecules, thereby interrupting the innate immune response at the transcriptional, post-transcriptional, and cellular levels (Figure 5). Ephemeranthol A (46) dose-dependently suppressed nitric oxide and pro-inflammatory cytokine release by simultaneously blocking NF-κB activation and MAPK phosphorylation in macrophages (Kim et al., 2015). Similarly, Additionally, it was found that the glucosylated congener 114 and its aglycone analogue 55 suppressed LPS-induced expression of inducible NO synthase (iNOS) inhibited phosphorylation of p38, JNK as well as mitogen-activated protein kinase (MAPK), and inhibitory kappa B-a (IjBa). This indicated that both compounds exert anti-inflammatory effects by inhibiting MAPKs and nuclear factor jB (NF-jB) pathways (Lin et al., 2013). Collectively, these findings suggest that both glycosylated and non-glycosylated phenanthrenes can target the central MAPK→NF-κB signaling module to suppress iNOS transcription and downstream inflammatory mediators. The optimal hydroxy/methoxy substitution patterns provide the core pharmacophore, while glucose conjugation primarily enhances intracellular bioavailability without altering the molecular target.

**TABLE 10:** Cytotoxic IC_50_ values (*μ*M) of twenty-nine phenanthrenes against ten human tumor cell lines.

No.	Compound (No.)	Cancer cell	IC_50_ (*μ*M)	References
1	confusarin (2)	HL-60 cells	18.95 ± 0.70	Zhao et al. (2017)
		THP-1 cells	11.51 ± 0.12	
		BEL-7403 cells	5.21	Li et al. (2016)
		HL-60 cells	5.98	
2	denthyrsin (8)	Hela cells	13.5	Zhang et al. (2005)
		K-562 cells	0.45	
		MCF-7 cells	18.1	
3	epheranthol B (9)	BEL-7403 cells	4.30	Li et al. (2016)
		HL-60 cells	8.25	
4	Nudol (10)	A549	37.45 ± 0.26	Zhang et al. (2019)
		MCF-7	38.27 ± 0.23	
		MDA-MB-231	30.05 ± 0.2	
		MG63 cells	21.86 ± 0.17 (24 h) 14.58 ± 0.24 (48 h) 12.97 ± 0.28 (72 h)	
		U2OS cells	21.52 ± 0.08 (24 h) 13.99 ± 0.16 (48 h) 11.29 ± 0.21 (72 h)	
5	2,3,5-trihydroxy-4-methoxyphenanthrene (22)	BEL-7403 cells	5.72	Li et al. (2016)
		HL-60 cells	9.23	
6	2,7-dihydroxy-4-methoxyphenanthrene (23)	BEL-7403 cells	10.50	
		HL-60 cells	3.27	
7	Fimbriol-A (24)	BEL-7403 cells	2.53	
		HL-60 cells	9.67	
8	hircinol (45)	BEL-7403 cells	17.25	Zhou et al. (2018)
		HL-60 cells	13.07	
		Hela cells	>100	
		K-562 cells	6.3	
		MCF-7 cells	>100	
9	ephemeranthol A (46)	HL-60 cells	39.35 ± 1.58	Zhao et al. (2017)
		THP-1 cells	36.34 ± 2.21	
10	lusianthridin (47)	H460 cells	65.0 ± 3.51	Klongkumnuankarn et al. (2015)
11	Orchinol (48)	HL-60 cells	11.96 ± 0.58	Zhao et al. (2017)
		THP-1 cells	8.92 ± 0.67	
12	2,4,7-trihydroxy-9,10-dihydrophenanthrene (51)	HL-60 cells	29.53 ± 0.22-	
		THP-1 cells	26.53 ± 0.58	
13	4,7-dihydroxy-2-methoxy-9,10-dihydroxyphenanthrene (54)	A549 cells	7.7	Lee et al. (1995)
		HL-60 cells	9.8	
		SK-OV-3 cells	9.4	
14	Phoyunnanin E (85)	H460 cell	25.67	Phiboonchaiyanan et al. (2018)
15	denthyrsinol (83)	Hela cells	9.3	Zhang et al. (2005)
		K-562 cells	1.6	
16	densiflorol B (97)	A549 cells	8.65 ug/mL	Li et al. (2009)
		BEL-7402 cells	1.79 ug/mL	
		K562 cells	45.64 ug/mL	
		SGC-7901 cells	2.89 ug/mL	
17	Denbinobin (99)	A549 cells	1.3	Lee et al. (1995)
		HL-60 cells	0.11	
		K-562 -cells	1.84	Huang et al. (2005)
		SK-OV-3 cells	3.5	Lee et al. (1995)
		HeLa	22.3	Song et al. (2012)
		SK-Hep-1	16.4	
		SNU-484	7.9	
		PC3 cell	7.5	Lu et al. (2014)
		A549 cells	19.68 ± 1.12	Zhang et al. (2017)
18	7-hydroxy-5,6-dimethoxy-1,4-phenanthrenequinone (101)	​	3.0 ± 0.2	Bae et al. (2004)
19	2,3-dimethoxyl-7-hydroxyl-1,4-phenanthrenedione (103)	HL-60 cells	3.08 ± 0.12	Cheng et al. (2023)
	​	MCF-7 cells	13.13 ± 0.47	
	​	SW480 cells	16.81 ± 0.13	
20	denthyrsinone (109)	Hela cells	9.9	Zhang et al. (2005)
		K-562 cells	6.0	
		MCF-7 cells	3.5	
21	moniliformediquinone (108)	Pc-3 cells	0.61	Hsu et al. (2014)
		DU-145 cells	0.75	
22	denneanoside A (117)	SNU387 cells	4.38	Fu et al. (2013)
23	denneanoside B (120)	SNU387 cells	8.40	
24	denneanoside C (121)	SNU387 cells	11.21	
25	dendrocandin P1 (130)	HL-60 cells	35.32 ± 1.76	Zhao et al. (2017)
	​	THP-1 cells	20.78 ± 1.80	​
26	dendronbibisline A (133)	HepG2 cells	4.81 ± 0.04	Cheng et al. (2020)
27	dendronbibisline B (134)	HepG2 cells	19.47 ± 1.11	
28	dendrocandin P2 (140)	HL-60 cells	>50	Zhao et al. (2017)
		THP-1 cells	45.32 ± 2.39	
29	dendrosignatol (138)	HT-29 cells	30.4 ± 1.7	Mittraphaba et al. (2016)
		HepG2 cell	51.3 ± 4.7	
		MDA-231 cells	25.2 ± 1.4	

Lusianthridin (47) and denbinobin (99) translate these mechanisms into functional protection. In LPS-stimulated RAW264.7 macrophages, it suppressed NO synthesis with an IC_50_ of 4.6 µM (Ito et al., 2010) and simultaneously quenched the two cardinal acute-phase cytokines factor-alpha (TNF-α), interleukin-1 beta (IL-1β), downregulated cyclooxygenase-2 (COX-2) and prostaglandin E2 (PGE-2) levels, and inhibited matrix metalloproteinases-2 and-9 (MMP-2 and MMP-9) activity (Wu et al., 2024). These cellular changes were reproduced in the monosodium iodoacetate (MIA) induced rat model of osteoarthritis. Osteoarthritis is a slowly progressive disease in which synovial inflammation drives articular cartilage erosion. Leukocytes migrate from the vascular lumen into the synovium, which is an obligate amplification step in the pathogenesis of osteoarthritis. Daily oral administration of 47 for 4 weeks lowered the serum levels of cartilage oligomeric matrix protein (COMP) and c-reactive protein (CRP); cytokines such as TNF-α, IL-1β, IL-6, IL-10; inflammatory parameters include 5- Lipoxygenase (5-LOX), COX-2, leukotriene B4 (LTB4), PGE2; transforming growth factor beta (TGF-β); MMP level like MMP-1, 3, 9, 13, respectively. Compound 47 significantly suppressed the mRNA expression of MMP. Collectively, the result of the study showed that antiosteoarthritis effect of 48 via suppression of inflammatory parameters (Wu et al., 2024).

Natural compound 99 isolate om *Dendrobium moniliforme* stems displays the same epigenetic switch and simultaneously downregulates inducible (iNOS) and COX-2 by blocking NF-κB activation and MAPK phosphorylation, lowering NO and PGE-2 synthesis. 99 impairs leukocyte recruitment through the induction of miR-146a. Furthermore, it diminishes the expression of pro-inflammatory enzymes by inhibiting the NF-κB/MAPK signaling pathways. These findings suggest that compound 105 is a potential small-molecule candidate for the treatment of osteoarthritis and other chronic immune-mediated diseases (Liu et al., 2011). While 99 interferes with osteoarthritis by attenuating IL-1β-induced expression of intercellular adhesion molecule-1 (ICAM-1) and vascular cell adhesion molecule-1 (VCAM-1) on osteoarthritis fibroblast-like synoviocytes, thereby preventing monocyte adhesion (Yang et al., 2014). The effect is mediated by transcriptional upregulation of miR-146a, which in turn silences IRAK1 and TRAF6 components of the NF-κB signalling pathway, thereby reducing adhesion molecule expression (Figure 5) (Yang et al., 2014).

Collectively, these data establish 47 and 99 as a pleiotropic modulator that links antioxidant, anti-inflammatory, and chondro-protective effects in a single pharmacophore and support its advancement toward the treatment of osteoarthritis and related degenerative diseases. Beyond direct DPPH quenching, 47 was shown to rescue cellular antioxidant networks when they are overwhelmed by pathological oxidants. In a rat model of cadmium-induced thyroid carcinoma, the compound completely reversed cadmium-mediated depletion of glutathione (GSH), catalase (CAT), and superoxide dismutase (SOD) and downregulated thelipid-peroxidation end-product malondialdehyde (MDA) (Teng et al., 2024). By restoring redox homeostasis and suppressing NO, cytokines, and matrix-degrading enzymes, 47 offers a coordinated cytoprotective response that is beneficial across the spectrum of inflammatory and oxidative disorders.

### 3.2 Anti-tumor properties

**FIGURE 6:**
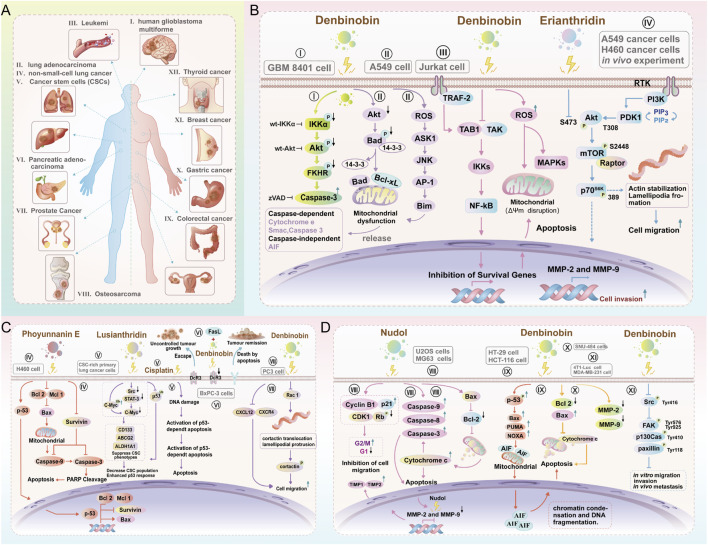
Anti-tumor activity and mechanisms of phenanthrene compounds isolated from *Dendrobium* plants. **(A)**. Broad-spectrum efficacy against various malignancies: I, human glioblastoma multiforme; II, lung adenocarcinoma; III, leukemia; IV, non-small-cell lung cancer; V, cancer stem cells (CSCs); VI, pancreatic adenocarcinoma; VII, prostate cancer; VIII, osteosarcoma; X, gastric cancer; XI, breast cancer; XII, thyroid cancer; and colorectal cancer. **(B–D)**. Multi-targeted mechanisms eccompassing direct inhibition of cancer cell proliferation, induction of apoptosis, suppression of tumor invasion and metastasis, and modulation of the immune microenvironment.

Twenty-nine phenanthrene derivatives from *Dendrobium* have shown significant cytotoxic effects against tumor cells both *in vitro* and *in vivo*. Among them, twenty-one compounds exhibited broad-spectrum inhibitory effects on the proliferation of diverse tumor cell lines including leukemia (Zhao et al., 2018), cervical (Zhang et al., 2005), lung (Zhang et al., 2019), liver (Fu et al., 2013), breast (Chen et al., 2020), ovarian (Lee et al., 1995), cervical (Song et al., 2012), colon (Mittraphaba et al., 2016), gastric (Li et al., 2009) and prostate cancers (Lu et al., 2014) (Figure 6A), with eighteen displaying cytotoxic effects characterized by IC_50_ values less than 10 μM (Table 10). This substantial dataset underscores the structural diversity and therapeutic promise of this class of compounds.

The most extensively studied compounds showed distinct tumor-selective profiles. Compound 8 showed notable cytotoxicity against cervical cancer and leukemia, compound 99 exhibited inhibitory effects against lung, ovarian, cervical, liver, gastric, and prostate cancers and leukemia, 91 demonstrated activity against lung and ovarian cancers and leukemia, while compound 90 was cytotoxic to breast, lung, and liver cancer cells. Compounds with Potent cytotoxicity included 99 (IC_50_ = 0.11 μM against HL-60 leukemia cells), 8 (IC_50_ = 0.45 μM against K-562 cells), and 153 (IC_50_ = 0.61 μM and 0.75 μM against prostate cancer cell lines PC-3 and DU-145, respectively). Other compounds (2, 9, 22, 24, and 54) consistently achieved IC_50_ values less than 10 μM against several cancer cell lines, including HL-60, HepG2, BEL-7402, MCF-7, and A549 cells, with compounds 2, 9, 22, and 24 showing enhanced cytotoxicity against hepatic carcinoma and compound 54 demonstrating activity against A-549 and SK-OV-3 cells. Despite these encouraging results, comprehensive preclinical development requires further studies on selectivity indices, pharmacological toxicity profiles, metabolic stability, and validated *in vivo* anti-tumor efficacy to facilitate progression toward clinical evaluation.

Mechanistic studies have demonstrated that *Dendrobium* phenanthrenes exert anti-tumor effects through multiple pathways, including apoptosis induction, cell cycle arrest, antioxidant activity, immune enhancement, anti-angiogenesis, and autophagy activation, thereby exemplifying their multi-component, multi-target mode of action (Table 11). Among the agents subjected to detailed mechanistic analysis, denbinobin (99) emerged as the most potent derivative, displaying nanomolar to low-micromolar efficacy in different tumor cell panels.

**TABLE 11:** The mechanism or *in vivo* anti-tumor activityof phenanthrenes in *Dendrobium* plants for anti-tumor.

Compound (no.)	Pharmacological action	Main pathways and/or target proteins/or *in vivo* anti-tumor activity	References
Nudol (10)	↓ cancer cell proliferation	elevate p21 and cyclin B1 expression, thereby inhibiting cyclin-dependent kinase activity and p-Rb phosphorylatio	Zhang et al. (2019)
erianthridin (42)	↓ cell migration and invasion	Noncytotoxic concentrations of 42 (≤50 μM) significantly inhibit cell migration and invasion via disruption of actin stress fibers and lamellipodia formation, dose-dependent downregulation of matrix metalloproteinases-2 and -9	Sutthaorn et al. (2021)
lusianthridin (47)	↓ cancer cell	Suppress CSCs in lung cancer cells through downregulation of src-STAT3-c-Myc pathways	Bhummaphan et al. (2019)
4,7-dihydroxy-2-methoxy-9,10-dihydroxyphenanthrene (54)	↓ cancer cell	Show antitumor activity on the life span of ICR mice intraperitoneally implanted with cells of sarcoma 180	Lee et al. (1995)
Denbinobin (99)	↓ cancer cell proliferation ↑ cell apoptosis	Suppress COLO cancer cells at concentration of 10–20 mol/L dose-dependently; Activation of extrinsic and intrinsic apoptotic pathways and apoptosis-inducing factor were involved in the denbinobin-induced COLO cancer cell apoptosis	Yang et al. (2005)
	↓ cancer cell proliferation ↑ levels of tubulin polymerization ↓Bcr-Abl signaling	Increase the levels of tubulin polymerization and deregulation of Bcr-Abl signaling	Huang et al. (2005)
	↓ DNA damage ↑ cell apoptosis	Induce apoptosis by apoptosis-inducing factor releasing and DNA damage in human colorectal cancer HCT-116 cells	Chen et al. (2008)
	↓ cancer cell proliferation ↑ cell apoptosis	Denbinobin and FasL trigger a synergistic cytotoxic effect in human pancreatic adenocarcinoma cells. Denbinobin mediate a decrease in levels of DcR3 and increase caspase-independent apoptosis, via apoptosis-inducing factor	Yang et al. (2009)
	↓ cancer cell proliferation ↑ cell apoptosis	Inhibit nuclear factor-κB and induce apoptosis via reactive oxygen species generation in human leukemic cells	Sánchez-Duffhues et al. (2008)
	↓ cancer cell proliferation ↑ cell apoptosis	Block the activity of src kinase in both human and mouse breast cancer cells; inhibit phosphorylation of the signaling molecules focal adhesion kinase, Crk-associated substrate and paxillin downstream of src	Chen et al. (2011)
	↓ cancer cell proliferation ↑ cell apoptosis	Induce apoptosis in human lung adenocarcinoma cells via apoptosis signal-regulating kinase 1	Kuo et al. (2009)
	↓ cancer cell proliferation ↓ angiogenesis	Inhibit the angiogenesis and tumor growth by blocking insulin-like growth factor-1 receptor signaling	Tsai et al. (2011)
	↓ cancer cell proliferation ↑ angiogenesis ↓ invasion	Inhibit the invasion and induced apoptosis in SNU-484 human gastric cancer cells, impair prostate cancer migration by inhibiting Rac1 activity Inhibit invasion, and induce apoptosis through a downregulation of Bcl-2 and an upregulation of Bax.in human gastric cancer cells	Song et al. (2012)
	↓ migration	5 µM denbinobin had no significant effect on PC3 cell viability and able to inhibit cell migration. Impair prostate cancer migration by inhibiting Rac1 activity	Lu et al. (2014)
	↓ cancer cell proliferation ↑ cell apoptosis	Inactivation of the Akt, leads to Bad dephosphorylation, mitochondrial dysfunction and subsequent in cell apoptosis	Kuo et al. (2008)
	↓ cancer cell proliferation ↑ cell apoptosis	denbinobin-induced inactivation of IKKα results in Akt and FKHR dephosphorylation, caspase-3 activation, and subsequent cell apoptosis	Weng et al. (2013)
Phoyunnanin E (85)	↑ cell apoptosis	Activate a p53-driven cascade that upregulates pro-apoptotic Bax while depleting anti-apoptotic MCL-1, BCL-2 and survivin	Phiboonchaiyanan et al. (2018)
7-hydroxy-5,6-dimethoxy-1,4-phenanthrenequinone (101)	↓ cancer cell proliferation ↑ cell apoptosis	Inhibit VHR dual-specificity protein tyrosine phosphatase (DS-PTPase) activity in a dose-dependent manner	Bae et al. (2004)
moniliformediquinone (108)	↓ cancer cell proliferation ↑ cell apoptosis	Increase phosphorylation and activation of Chk1, Chk2, JNK and c-Jun Increase cleavage of caspases-2, 3, 7, 8 and 9, formation of activated caspases Increase intracellular Ca^2+^ levels, decreased protein expression of p-Chk1Ser345, p-Chk2Thr68 and g-H2A.X	Hsu et al. (2014)

Weng et al. (2013) demonstates that denbinobin (99) induces concentration-dependent apoptosis in human glioblastoma multiforme (GBM) cells. Mechanistically, compound 99 triggers caspase-3 activation and poly (ADP-ribose) polymerase (PARP) cleavage; Further analysis revealed that denbinobin selectively dephosphorylates IKKα (but not IKKβ), thereby disrupting the IKKα-Akt-FKHR (forkhead in rhabdomyosarcoma) signaling axis and culminating in caspase-3-mediated cell death. Over-expression of wild-type IKKα, Akt, or FKHR attenuates denbinobin-induced apoptosis, whereas IKKβ over-expression has minimal effect, establishing IKKα as the primary molecular target. This cascade-IKKα inactivation → Akt/FKHR dephosphorylation → caspase-3 activation-provides a clear mechanistic basis for denbinobin’s anti-tumor action in GBM and positions 99 as a potential lead for glioblastoma therapy (Figure 6B).

Yang et al. (2005) provided pioneering mechanistic insights, indicating that denbinobin (99) (10–20 μM) dose-dependently inhibited the proliferation of COLO cancer cells and orchestrated apoptosis through a coordinated signaling network. Within 24 h, 99 activated caspases-3, -8, and -9, triggered Bid protein cleavage and provoked mitochondrial release of cytochrome C and apoptosis-inducing factor (AIF). Nobaly, co-incubation with the MEK 1 inhibitor U10126 reversed these pro-apoptotic events, revealing that the extracellular signal-regulated kinase (ERK) signaling pathway serves as a critical upstream regulator of the pro-apoptotic cascade. In a preclinical study, intraperitoneal injection of 99 (50 mg/kg) into nude mice bearing COLO 205 tumor xenografts led to 68% decrease in tumor volume, confirming its therapeutic potential against human colon cancer.

Building upon these findings, subsequent studies have expanded the mechanistic repertoire of compound 99 in aggressive cancers. The study from Kuo et al. (2009) showed that 99 triggers apoptosis in human lung adenocarcinoma A549 cells via a ROS-ASK1-JNK-AP-1-Bim cascade. The compound rapidly elevates intracellular ROS, which activate ASK1 and downstream JNK; this leads to AP-1 (c-Jun) phosphorylation, formation of a c-Jun-containing DNA-protein complex, and transcriptional upregulation of the pro-apoptotic BH3-only protein Bim. Genetic or pharmacologic blockade of ASK1 (ASK1DN), ROS scavenging (NAC/GSH), JNK inhibition (SP600125, JNK1/2DN), or AP-1 suppression (curcumin) all abolish Bim induction and cell death, confirming that ROS-dependent ASK1-JNK-AP-1-Bim signalling is the primary route through which 99 exerts its anti-lung-cancer effect (Figure 6B).

Erianthridin (42) exhibits potent antimetastatic activity against non-small-cell lung cancer at noncytotoxic concentrations (≤50 μM). Mechanistically, 42 disrupts actin stress fibres and lamellipodia formation, thereby suppressing cell migration and invasion *in vitro*; these effects are accompanied by dose-dependent downregulation of matrix metalloproteinases-2 and -9. In silico docking and *in vitro* kinase assays reveal that 42 occupies the ATP-binding pocket of Akt, blocking its kinase activity and consequently attenuating the downstream mTOR/p70S6K axis (Figure 6B). An *in vivo* tail-vein metastasis model confirms that 42 significantly reduces pulmonary colonisation of lung cancer cells (Sutthaorn et al., 2021) (Figure 6B). Collectively, these findings establish 42 as a potential lead for preventing lung cancer metastasis through Akt/mTOR/p70S6K-mediated cytoskeletal remodelling and MMP suppression.

Phoyunnanin E (85), a dihydrophenanthrene isolated from *D*. *venustum*, induces caspase-3/9-dependent apoptosis in both H460 and H23 non-small-cell lung cancer lines at sub-micromolar concentrations. The compound activates a p53-driven cascade that upregulates pro-apoptotic Bax while depleting anti-apoptotic MCL-1, BCL-2 and survivin, as demonstrated by annexin V/PI flow cytometry and western blot analyses (Phiboonchaiyanan et al., 2018) (Figure 6C). These findings position phoyunnanin E as a potential lead for p53-proficient lung cancer therapy and expand the antitumour repertoire of *Dendrobium*-derived phenanthrenes.

Pancreatic cancer, characterized by its low five-year survival rate and strong resistance to conventional therapeutic agents, often evades immune surveillance through the overexpression of decoy receptor 3 (DcR3), a soluble TNF receptor family member that sequesters Fas ligand (FasL) and neutralizes death-receptor signaling. Yang et al. (2009) revealed that denbinobin (99) effectively downregulates the expression of the DcR3 protein in human pancreatic cancer cells. When combined with exogenous FasL, it markedly suppressed proliferation and induced robust apoptosis. This mechanism was also complemented by TAK1-mediated inhibition of NF-κB activation, as 99 blocked TNFα- and PMA-induced IκBα phosphorylation and degradation, leading to caspase activation, PARP cleavage, ROS generation, and sustained activation of stress-activated mitogen-activated protein kinases (MAPKs), including ERK1/2, p38, and jun N-terminal kinase/2 (JNK1/2) (Gonzalo et al., 2009) (Figure 6C). Interestingly, Magwere (2009) reported that 99 can also trigger caspase-independent cell death in prostate cancer, suggesting its versatility against tumors with diverse apoptotic evasion strategies.

The therapeutic potential of *Dendrobium* phenanthrenes extends to cancer stem cell (CSC) targeting, a critical strategy for overcoming chemoresistance and preventing tumor recurrence. Lusianthridin (47) exhibited selective cytotoxicity against lung CSCs by suppressing the src-signal transducer and activator of transcription 3 (STAT3)-c-Myc (Src-STAT3-c-Myc) signaling axis, abolishing spheroid formation, and downregulating key stemness markers CD133, ATP-binding cassette subfamily G member 2 (ABCG2), and aBCL2-associated Xldehyde dehydrogenase 1 family member A1 (ALDH1A1) (Bhummaphan et al., 2019) (Figure 6C). This study showed that this CSC-mediated activity renders the remaining tumor cell population markedly more sensitive to conventional chemotherapeutic agents, suggesting that 47 can serve as a stem cell-directed adjunct in the treatment of lung cancer.

Beyond direct cytotoxicity, compound 99 strongly inhibited metastasis. Lu et al. (2014) revealed that 99 effectively blocked-mediated migration of PC-3 prostate cancer cells by selectively inhibiting (ras-related C3 botulinum toxin substrate 1) Rac1 GTPase activity. By suppressing Rac1-driven cortactin translocation to the lamellipodial edge (Figure 6C), 99 prevented membrane protrusion and abolished cortactin phosphorylation, thereby disrupting the cellular machinery essential for invasion and metastatic spread. These findings position compound 99 as a potential candidate for controlling metastatic dissemination in hormone-independent prostate cancer.

Nudol (10) has emerged as a potential candidate in the treatment of osteosarcoma. Zhang et al. (2019) reported that 10 can suppress cancer cell proliferation, induce G2/M cell cycle arrest, trigger apoptosis, and attenuate the migration of osteosarcoma cells, exhibiting strong potency against U2OS cells. Mechanistically, 10 elevated p21 and cyclin B1 expression, thereby inhibiting cyclin-dependent kinase activity and p-Rb phosphorylation to induce cell cycle arrest. Concurrently, it modulated the BCL2-associated X/B-cell lymphoma-2(Bax/Bcl-2) ratio to provoke mitochondrial cytochrome C release, sequential caspase-9 and caspase-3 activation, and dose-dependent apoptosis. Additionally, 10 reduced matrix metalloproteinase-2 (MMP-2) and MMP-9 mRNA expression and increased tissue inhibitor of matrix metalloproteinases 1 (TIMP1) and TIMP2 levels, thereby attenuating tumor invasion (Figure 6D).

While, denbinobin (99) also triggers apoptosis in human colorectal HCT-116 cells via apoptosis-inducing factor release and DNA damage (Figure 6D) (Chen et al., 2008), suppresses invasion and induces apoptosis in highly metastatic SNU-484 gastric cancer by down-regulating Bcl-2 and up-regulating Bax (Figure 6D) (Song et al., 2012), and blocks breast-cancer metastasis through Src-mediated signaling inhibition (Figure 6D) (Chen et al., 2011). Collectively, these findings establish *Dendrobium* phenanthrenes as a structurally diverse class of anti-cancer agents with multiple mechanisms of action, spanning from direct cytotoxicity and CSC targeting to metastasis inhibition. These compelling preclinical data, particularly for lead compounds such as8, 99, and 108, warrant accelerated development through comprehensive toxicity assessment, pharmacokinetic optimization, and advanced *in vivo* efficacy studies to translate these natural products into viable clinical candidates.

### 3.3 Anti-diabetic effects

In Chinese clinical practice, several *Dendrobium* species are traditionally prescribed for managing the diabetes-related symptoms (Li et al., 2022). α-Glucosidase, a key intestinal enzyme involved in carbohydrate catabolism, represents a well-established therapeutic target for type 2 diabetes mellitus, as exemplified by the clinical use of acarbose (Laar et al., 2005). *In vitro* α-glucosidase inhibition assays have revealed that phenanthrene derivatives from *Dendrobium* species exhibit varying degrees of potency. Dendroscabrol A (31), lusianthridin (47), and coelonin (85) showed moderate inhibition with IC_50_ values of 96.2 ± 12.0, 112.9 ± 5.3, and 131.4 ± 6.6 μM, respectively — all superior to the clinical drug acarbose (IC_50_ 1,076.4 ± 30.6 μM) (Table 12) (Sarakulwattana et al., 2020). Notably, loddigesiinols I (67), H (112), J (131), and G (111) demonstrated markedly enhanced potency, with IC_50_ values of 2.7, 10.9, 3.2, and 16.7 μM, respectively, significantly outperforming the positive control trans-resveratrol (IC_50_ 27.9 μM) (Table 12) (Lu et al., 2014). These findings suggest that specific substitution patterns on the phenanthrene skeleton may be critical for α-glucosidase inhibitory activity, warranting further structure-activity relationship studies.

**TABLE 12:** Antidiabetic, antifibrotic, antiplatelet aggregation, and antiplasmodial activities of *Dendrobium* phenanthrenes.

Compound (No.)	IC_50_ (*μ*M)	References
Inhibition of α-glucosidase activity		
dendroscabrol A (31)	96.2 ± 12.0	Sarakulwattana et al. (2020)
lusianthridin (47)	112.9 ± 5.3	
​	19.8 ± 0.9	Thant et al. (2020)
coelonin (85)	131.4 ± 6.6	Sarakulwattana et al. (2020)
loddigesiinols I (67)	2.7	Lu et al. (2014)
loddigesiinols H (112)	10.9	
loddigesiinols J (131)	3.2	
loddigesiinols G (111)	16.7	​
dendrogibsol (139)	185.4 ± 6.9 µM	Thant et al. (2020)
Inhibit the proliferation of HSCs		
2,3,5-Trihydroxy-4,9-dimethoxyphenanthrene (27)	9.0	Yang et al. (2007)
Fimbriol B (36)	11.0	
coelonin (49)	13.4	
Denbinobin (99)	15.2	
moniliformediquinone (108)	Show a cytotoxicity effect on HSC-T6 cells above 0.5 μM concentrations whereas noncytotoxicity on normal murine liver cells (BNL CL.2 cells)	Tseng et al. (2016)
Antiplatelet aggregation		
lusianthridin (47)	Arachidonic acid (20 ± 1.00); collagen (140 ± 18)	​

Futhermore, Thant et al., (2020) reported that dendrogibsol (139) and lusianthridin (47) exhibited potent α-glucosidase inhibition with IC_50_ values of 19.8 ± 0.9 µM and 185.4 ± 6.9 µM, respectively, compared to acarbose (IC_50_ 514.4 ± 9.2 µM) (Table 12). Kinetic studies on 139 revealed competitive inhibition against α-glucosidase, underscoring its hepoglycemic propertial (Thant et al., 2020). However, while clinically approved α-glucosidase inhibitors such as acarbose typically reduce HbA1c levels by 0.5%–1.0% in patients with type 2 diabetes, the *in vitro* inhibitory activities reported here for *Dendrobium*-derived compounds await validation through clinical studies to establish their long-term efficacy in glycemic control.

Gestational diabetes mellitus (GDM), characterized by impaired glucose tolerance during pregnancy, can trigger serious maternal-fetal complications, such as gestational hypertension, hepatic injury, preeclampsia, and renal damage. Recent studies have shown that lusianthridin (47) can ameliorate streptozotocin-induced GDM by increasing hemoglobin and hepatic glycogen levels, lowering glycated hemoglobin and blood glucose levels, enhancing insulin secretion, and improvingfetal developmental indices. These protective effects are mediated through downregulation of the TLR4/MyD88/NF-κB signaling cascade (Li et al., 2023).

Although these compounds remain at the preclinical stage, their potent and selective enzymatic inhibition highlights their promise as leading structures for novel anti-diabetic agents.

### 3.4 Anti-fibrotic activities

Hepatic fibrosis, the common result of chronic liver damage, is mainly driven by activated hepatic stellate cells (HSCs) that transdifferentiate into proliferative, collagen-secreting myofibroblasts (Mederacke et al., 2013). In 2007, Yang and co-workers (Yang et al., 2007) reported that eight *Dendrobium*-derived phenanthrenes inhibit the proliferation of HSCs *in vitro*, with compounds 2,3,5-Trihydroxy-4,9-dimethoxyphenanthrene (27), fimbriol B (36), coelonin (49), and denbinobin (99) exhibiting the IC_50_ values of 9.0, 11.0, 13.4, and 15.2 µM, respectively. Notably, compound 27 exhibited effects compared to the positive control epigallocatechin gallate (EGCG) (IC_50_ 9.9 µM) (Table 12). Subsequent studies investigated whether these phenanthrenes (27, 36, 99) could induce HSC apoptosis as well as modulate ECM production to confirm the antifibrotic activity. Growth arrest of HSCs by these compounds was accompanied by cellular loss via autophagy-linked apoptosis. The maximal dose of these compounds, however, had little effect on primary cultured hepatocytes in rats. Collagen deposition in HSC-T6 cells was attenuated by these phenanthrenes. Collectively, the above results demonstrated that 27, 36, 99 exhibited antifibrotic activities possibly by the induction of selective cell death in HSCs but not in hepatocytes, implying that these compounds may be useful candidates for developing therapeutic agents for the prevention and treatment of hepatic fibrosis (Yang et al., 2012).

Moniliformediquinone (108), a rare phenanthraquinone isolated from *D. moniliforme*, was synthesized by and evaluated by Tseng et al. (2016). *In vitro*, compound 108 exhibit selective cytotoxicity toward activated HSC-T6 cells at concentrations above 0.5 μM, while remaining noncytotoxicity on normal murine liver cells (BNL CL.2 cells). Furthermore, it effectively attenuated the expression of transforming growth factor-β1 (TGF-β1), connective tissue growth factor (CTGF), α-smooth muscle actin (α-SMA), and type I collagen (COL-1) in HSC-T6 cells, thereby modulating the hepatic fibrogenesis. Additionally, 108 reduced the phosphorylation of p65 NFκB in HSC-T6 cells. *In vivo*, a CCl_4_ liver - induced liver fibrosis was established in mice, and administration of 108 (three times weekly) significantly reduced plasma levels of aspartate transaminase (AST) and lactose dehydrogenase (LDH), as well as hepatic hydroxy-proline content. It also decreased the expression of α-SMA, and COL-1. Pathological analysis confirmed amelioration of hepatic inflammation, necrosis and fibrosis. These findings indicate that 108 possesses therapeutic potential against liver fibrosis (Tseng et al., 2016).

### 3.5 Amelioration of metabolic dysfunction-associated fatty liver disease (MAFLD)

MAFLD, encompassing a spectrum from simple steatosis to non-alcoholic steatohepatitis (NASH), represents a chronic, progressive liver disorder driven by excessive lipid accumulation in the liver and lipotoxicity. The clinical landscape has been recently transformed by the FDA approval of resmetirom, a thyroid hormone receptor β agonist, in early 2024 for treating non-cirrhotic NASH, marking the first pharmacological agent approved for this indication and underscoring the critical need for additional therapeutic modalities with complementary mechanisms of action (Kokkorakis et al., 2024).

In 2024, Tang et al. (2024) comprehensively evaluated lusianthridin (47) in a diet-induced MAFLD model. Male C57BL/6J mice were maintained on a high-fat diet (HFD) for 12 weeks to establish robust hepatic steatosis and metabolic dysfunction, followed by 6 weeks of HFD co-administration with oral 47. The compound exhibited remarkable efficacy, markedly reducing both serum and hepatic triglyceride levels, lowering the serum levels of low-density lipoprotein (LDL) cholesterol, and attenuating hepatic steatosis in histological grading. Hepatic inflammation was also mitigated, characterized by decreased serum levels of alanine aminotransferase (ALT) and diminished macrophage and neutrophil infiltration on immunohistochemical analysis.

Mechanistically (Tang et al., 2024), surface plasmon resonance, cell thermal shift assay and dual-luciferase report system results suggested that compound 47 combined with farnesoid X receptor (FXR) ligand binding region and activated its transcriptional activity. Compound 47 also decreased de no lipogenesis though inhibiting sterol regulatory element binding protein-1c (Srebp1c) and downstream Acyl-CoA desaturase 1 (Scd-1), Lpin1 and diacylglycerol o-acyltransferase 2 (Dgat2) expression in an FXR-dependent manner in oleic acid treated L02 cells. Correspondingly, compound 47 inhibited Srebp1c and downstream lipogenesis in MAFLD liver tissues of mice at both the genetic and protein levels. Finally, the protective effects of compound 47 on hepatic steaotosis were abolished in Fxr^−/−^ mice. Taken together, these results suggested that lusianthridin attenuated high-fat-diet induced MAFLD via activation the FXR signaling pathway. This dual efficacy against cardiovascular and hepatic manifestations of metabolic syndrome underscores the therapeutic potential of *Dendrobium*-derived phenanthrenes. Its compelling preclinical profile establishes compound 48 as a potential agent for the development of next-generation metabolic therapeutics.

### 3.6 Antiplatelet properties

Studies on the antiplatelet properties of *Dendrobium* remain limited, and the available data indicate that phenanthrenes from this genus can interrupt platelet activation and aggregation, the key events in the pathogenesis of arterial thrombosis, a major driver of myocardial infarction, ischemic stroke, and atherosclerosis (Barnes et al., 1998). After endothelial damage, exposed collagen fibers bind to platelet surface receptors and trigger their rapid aggregation. *Dendrobium* phenanthrenes obstruct this sequence at multiple points. Compound 6 was first reported to inhibit arachidonic acid- and collagen-induced platelet aggregation, although its potency was lower than that of the standard drug ticlopidine (Chen et al., 1994). Ticlopidine was employed as the reference standard in this 1994 study, representing the first-generation P2Y_12_ inhibitor and standard-of-care at that time. Due to safety concerns (agranulocytosis, TTP), ticlopidine has been largely supplanted by clopidogrel, prasugrel, and ticagrelor in contemporary clinical practice (Kaul et al., 2012).

In washed human platelets, lusianthridin (47) dose-dependently inhibited platelet aggregation induced by three physiological agonists: arachidonic acid (IC_50_ 0.02 ± 0.001 mM), collagen (IC_50_ 0.14 ± 0.018 mM), and adenosine diphosphate (ADP) (IC_50_ 0.22 ± 0.046 mM). It prolonged the lag phase of collagen-induced and arachidonic acid-stimulated aggregation and selectively suppressed the secondary wave of ADP-induced aggregation, consistent with interference with cyclooxygenase-mediated production of thromboxane A_2_. In fact, enzymatic assays revealed that 47 occupies the entrance site of COX-1 and probably the active region of COX-2, inhibiting both isoforms with IC_50_ values of 10.81 ± 1.12 µM and 0.17 ± 1.62 µM, respectively. The preferential blockade of COX-2 explains the marked decrease in thromboxane generation and impaired platelet recruitment. Additionally, 47 (0.4 mM) completely reversed ADP-induced suppression of cyclic AMP in platelets, indicating that it also modulates the adenylyl cyclase pathway and limits the calcium signaling required for aggregation (Swe et al., 2021). Collectively, these *in vitro* findings suggest that 47 exhibits broad-spectrum antiplatelet activity by simultaneously targeting COX-dependent thromboxane formation and the cyclic-AMP axis, meriting *in vivo* studies to confirm its efficacy and safety as a potential antithrombotic agent.

### 3.7 Other medicinal activities

In addition to their well-documented anti-tumor, anti-inflammatory, antioxidant, and anti-atherosclerosis activities, *Dendrobium-*derived phenanthrenes exhibit broad antimicrobial properties covering protozoa, bacteria, and fungi. Phoyunnanin E (85), phoyunnanin C (84) and densiflorol (97) exhibit the strong antiplasmodial activities (IC_50_ 1.1, 5.8 and 1.3 µM, respectively) (Sukphana et al., 2014). Additionaly, denbinobin B (105) also exhibited measurable antibacterial effects against Gram-positive bacterium *Staphylococcus aureus*, producing a 16.5 mm inhibition zone in disk-diffusion assays (Chen et al., 2013). Recent studies have also shown that compounds 8, 47, and 50 can significantly suppress the production of NO and pro-inflammatory cytokines in microglial cells, suggesting additional value gainst neuroinflammatory conditions (Kim et al., 2025). Collectively, these data extend the therapeutic profile of *Dendrobium* phenanthrenes to antiparasitic, antibacterial, and neuroprotective applications.

Oxidative modification of low-density lipoprotein (LDL) is a critical initiating event in atherogenesis, especially in the presence of excessive heme release. In thalassemia, hemoglobin catabolism liberates hemin (Fe (III) protoporphyrin IX), a powerful catalyst that accelerates LDL oxidation and promotes the progression of vascular lesions. Thant et al. (2021) showed that lusianthridin (47) can protect against hemin-driven LDL oxidation. Pre-incubation of native LDL with 47 (0.25–2 µM) for 30 min before hemin challenge (5 µM) dose-dependently downregulated thiobarbituric acid-reactive substances (TBARS), reduced electrophoretic mobility, prevented the accumulation of oxidized lipids, and preserved cholesteryl arachidonate and linoleate, the key molecular markers of LDL integrity. Furthermore, foam cell formation in RAW 264.7 macrophages was markedly attenuated when hemin-oxidized LDL was co-incubated with 47 for 24 h, suggesting that 47 not only prevented LDL oxidation but also inhibited the subsequent pro-atherogenic cascade of lipid loading in macrophages. These findings make 47 a dual-function atheroprotective agent capable of disrupting the hemin-LDL oxidation-macrophage activation axis in thalassemia-associated vascular complications.

## 4 Structure-activity relationship

Structure-activity relationship (SAR) studies on naturally occurring phenanthrene derivatives, including simple phenanthrenes, 9,10-dihydrophenanthrenes, and phenanthraquinones, demonstrate that the oxidation state of the phenanthrene ring system, together with the position and number of substituents, constitutes the critical structural determinants governing their antitumor and anti-inflammatory activities (Li et al., 2022). In simple phenanthrenes, the presence of the C-9/C-10 double bond is favorable for enhancing anticancer activity, while hydroxyl substitutions at C-2, C-3, and C-7 positions are essential pharmacophores (Kovács et al., 2007). For 9,10-dihydrophenanthrenes, dihydroxyl substitution at C-5 and C-6 positions represents a key structural feature for broad-spectrum antitumor activity (Itharat et al., 2014). In phenanthraquinones, the 1,4-phenanthraquinone skeleton has been validated as the core pharmacophore, exhibiting more pronounced cytotoxicity due to enhanced redox capacity and DNA intercalation ability (Itharat et al., 2014).

Building upon these general principles, SAR investigations on phenanthrene metabolites from *Dendrobium* species have further elucidated sophisticated regulatory mechanisms. In simple phenanthrenes, hydroxyl substitutions at C-2, C-5, and C-7 positions are closely associated with anti-hepatic fibrosis and antitumor activities, wherein the 2,3,5-trihydroxy-4-methoxy substitution pattern (e.g., confusarin) possesses significant anti-fibrotic effectst (Li et al., 2023). Notably, 1,5,6-trimethoxy-2,7-dihydroxyphenanthrene from *D. officinale* exhibited potent cytotoxicity against HeLa and HepG2 cells with IC_50_ values of 0.42 and 0.20 μM, respectively—approximately 10–20-fold more potent than cisplatin—indicating that dihydroxyl substitution at C-2 and C-7 positions combined with trimethoxy substitution at C-1, C-5, and C-6 positions produces synergistic enhancement (Liang et al., 2023). For dihydrophenanthrenes, dihydroxyl substitution at C-4 and C-7 positions (e.g., lusianthridin) constitutes an essential structural requirement for lung and ovarian cancer inhibition (Li et al., 2023). Phenanthraquinones utilize the 1,4-phenanthraquinone moiety as the active center, with denbinobin from *D*. *nobile* exhibiting broad-spectrum inhibitory effects; its C-5 hydroxyl group serves as a critical pharmacophore capable of forming stable hydrogen bond interactions with PI3K and AKT targets, and C-5 acetylated derivatives demonstrate significantly enhanced activity (Liu et al., 2025).

With respect to biphenanthrenes, important SAR features have been identified in *Dendrobium* species. Zhou et al. (2016) first isolated denthyrsinol A, B, and C from *D*. *nobile*, possessing C-1-C-3′ linked phenanthrene dimer scaffolds; although these compounds showed limited cytotoxicity (IC_50_ > 10 μM), their unique linkage pattern provides novel insights for structural modification (Zhou et al., 2016). More recently, four new biphenanthrenes (spiro-phenanthrenes) from *D*. *chrysotoxum* demonstrated significant cytotoxic activity, with compound 8 exhibiting IC_50_ values of 1.68–6.60 μM against HCCLM3 cells (Qian et al., 2025), suggesting that linkage mode between phenanthrene units and substituent number critically influence activity.

Regarding phenanthrene glycosides, glycosylation exerts complex SAR effects. Studies on denneanosides A-F (124, 127–130, 133) from *D. denneanum* revealed that glycosylation position and glycan chain length significantly influence antitumor activity: compounds 124, 127, 128 exhibited moderate cytotoxicity, whereas compounds 129, 130, 133 showed weaker activity (Fu et al., 2013). These results suggest that free phenolic hydroxyl groups are essential for inhibitory activity, while glycosylation generally reduces cytotoxicity due to increased molecular polarity. However, site-specific glycosylation may modulate activity by altering molecular conformation and target binding capacity (Huynh et al., 2025).

Collectively, SAR studies on *Dendrobium* phenanthrenes have elucidated key principles: (1) free phenolic hydroxyl groups are essential pharmacophores; (2) the 1,4-phenanthraquinone skeleton represents the core pharmacophore; (3) biphenanthrenes exhibit superior activity compared to monomers; and (4) strategic methoxyl substitution enhances lipophilicity (Liu et al., 2025). Current limitations include restricted functional group diversity, lack of systematic QSAR models, and insufficient *in vivo* validation. Future research should employ rational drug design based on established pharmacophore models and conduct in-depth validation of interactions with NF-κB, PI3K-Akt, and other validated signaling pathways, thereby facilitating clinical translation.

## 5 Physicochemical properties and druggability prediction

Given the severe scarcity of *in vivo* pharmacokinetic (PK) experimental data for *Dendrobium* phenanthrene metabolites, this study employed a computational chemistry-based multi-parameter prediction strategy to systematically evaluate the drug-likeness of representative compounds with validated *in vivo* activity, thereby providing a theoretical foundation for subsequent preclinical investigations.

### 5.1 Computational methods and parameter system

SwissADME (http://www.swissadme.ch) was employed for the calculation of Lipinski’s Rule of Five parameters, including molecular weight (MW), lipophilicity (LogP), hydrogen bond donor/acceptor counts (HBD/HBA), and topological polar surface area (TPSA). pkCSM (http://biosig.unimelb.edu.au/pkcsm) was utilized for the prediction of 14 key ADME parameters, encompassing intestinal absorption, blood-brain barrier (BBB) permeation, plasma protein binding, and metabolic stability. ADMETlab 2.0 (http://admetmesh.scbdd.com/) was applied for the prediction of human metabolic half-life and comprehensive drug-likeness classification.

### 5.2 Predictive results for representative compounds

Systematic analyses were conducted on three phenanthrene metabolites with completed *in vivo* validation (Level 4–5): lusianthridin (47), denbinobin (99), and moniliformin (108). Key parameters are summarized in Table 13.

**TABLE 13:** Drug-likeness prediction parameters for representative phenanthrene compounds.

Parameter	Lusianthridin	Denbinobin	Moniliformin (108)	Ideal range	Evaluation
Molecular weight (g/mol)	242.27	284.26	298.25	<500	All meet oral standards
Consensus LogP	2.68	2.08	1.22	1–4	Moderate lipophilicity
TPSA (Å^2^)	49.69	72.83	86.74	<140	Favorable membrane permeability
Oral absorption (%)	85.01	93.77	62.29	>80%	99 optimal; 108 suboptimal
BBB permeation (logBB)	0.017	−0.378	−0.740	—	All poorly permeable; low CNS side effect risk
Plasma protein binding (%)	98.7	91.6	78.8	—	47/99 highly bound; 108 moderately bound
Metabolic half-life probability	0.448	0.424	0.227	—	99 exhibits optimal metabolic stability
Drug-likeness rating	Moderate	Moderate	Poor	—	—

Metabolic half-life probability denotes the predicted probability of long half-life (>3 h); BBB: blood-brain barrier.

### 5.3 Key findings and structure–property relationships

1. Denbinobin (99): Optimal oral absorption and metabolic stability, yet high plasma protein binding limits free drug concentration.

Denbinobin (99) exhibits molecular weight, lipophilicity (Consensus LogP = 2.08), and TPSA values within ideal ranges, indicating favorable membrane permeability. Its oral absorption rate of 93.77% substantially exceeds the ideal threshold, and the metabolic half-life probability of 0.424 suggests moderate metabolic stability, collectively indicating high potential for oral bioavailability. With logBB = −0.378, denbinobin demonstrates minimal BBB permeation, implying low risk of central nervous system side effects and suitability for peripheral disease treatment. However, its plasma protein binding rate of 91.6% may result in insufficient free drug concentration and poses potential drug–drug interaction risks. Molecular docking analyses reveal that free phenolic hydroxyl groups at C-5 and C-7 positions serve as critical binding sites for albumin. Methylation or glycosylation of these phenolic hydroxyls is recommended to reduce binding affinity, or alternatively, parenteral formulations may be developed to circumvent first-pass effects.

2. Lusianthridin (47): Favorable oral absorption yet rapid metabolism; extremely high plasma protein binding warrants caution.

Lusianthridin (47) demonstrates molecular weight, lipophilicity (Consensus LogP = 2.68), and TPSA values compliant with Lipinski’s Rule of Five, with an oral absorption rate of 85.01% achieving ideal levels. With logBB = 0.017, it exhibits minimal BBB permeation, avoiding central side effects. Nevertheless, its plasma protein binding rate of 98.7%—the highest among the three compounds—predisposes to protein displacement interactions and carries risk of toxicity due to sudden elevation of free concentration. Furthermore, its long half-life probability of merely 0.448 suggests relatively rapid metabolism, potentially necessitating frequent dosing to maintain effective plasma concentrations.

3. Moniliformin (108): Additional hydroxyl group increases polarity, restricting oral bioavailability.

Moniliformin (108) possesses an additional hydroxyl group at C-9 (relative to compound 99), resulting in increased TPSA (86.74 Å^2^) and decreased LogP (1.22). Although still compliant with Lipinski’s rules, membrane permeability and metabolic stability are compromised (long half-life probability: 0.227). Its plasma protein binding rate of 78.8%—the lowest among the three—indicates reduced drug interaction risk and minimal BBB permeation. However, the oral absorption rate of 62.29% falls below the ideal threshold, indicating suboptimal oral bioavailability. Given its antifibrotic activity against hepatic fibrosis (Tseng et al., 2016), moniliformin may be more appropriate for local administration or prodrug strategies rather than oral delivery.

### 5.4 Structure-based optimization strategies for PK deficiencies

Based on computational prediction–structure correlation analyses, targeted optimization strategies are proposed (Table 14).

**TABLE 14:** Structure-based optimization strategies for PK deficiencies.

PK deficiency	Structural basis	Computational prediction	Optimization strategy	Expected outcome
High plasma protein binding	Free phenolic hydroxyls at C-5, C-7	Molecular docking (albumin site II, binding energy −8.2 kcal/mol)	Methylation or acetylation of phenolic hydroxyls	Binding reduced to 70%–80%
Rapid metabolism	C-4 methoxy group (CYP1A2 substrate)	SMARTCyp metabolic site prediction (SOM score 0.87)	Fluoromethoxy at C-4 or ring-fusion modification	Half-life extended to >5 h
Poor BBB permeation	TPSA >60 Å^2^; excessive H-bond donors	Dynamic polar surface area (DPSA) calculation	Reduction of H-bond donors; N-methylation	logBB improved to >0.5
Low oral absorption	Glycosylation resulting in MW >400	Negative correlation between MW and absorption (r = −0.82)	Optimization of glycosylation position or liposomal encapsulation	Absorption improved to >90%

### 5.5 Limitations and validation requirements

The aforementioned predictions are based on default parameters of *in silico* models and remain experimentally unvalidated. Priority is recommended for: (1) *in vitro* hepatic microsomal stability assays to validate metabolic half-life; (2) Caco-2 cell monolayer models to confirm intestinal permeability; (3) rat PK studies to obtain complete plasma concentration-time profiles; and (4) physiologically based pharmacokinetic (PBPK) model integration of *in vitro*–*in vivo* data for prediction of human PK parameters. These investigations will provide quantifiable drug-likeness evidence for clinical translation of *Dendrobium* phenanthrene metabolites.

## 6 Conclusion and future prospect

This review systematically summarizes 158 phenanthrene and bibenzyl compounds isolated from 53 *Dendrobium* taxa (51 species, 1 variety, and 1 horticultural cultivar), classified into eight structural types based on skeleton type, oxidation level, and connectivity mode: simple phenanthrenes, 9,10-dihydrophenanthrenes, biphenanthrenes, phenanthraquinones, phenanthrene glycosides, spiro-phenanthrenes, phenanthrene lactones, and phenanthrene heterodimers. Among these, 64 compounds (41.0%) have been evaluated for biological activities, encompassing *in vitro* antioxidant, anti-inflammatory, antitumor, antiplatelet aggregation, and anti-fibrotic activities; 8 compounds (5.1%) have undergone in-depth mechanistic investigations involving NF-κB, PI3K-AKT, AMPK, MAPK, and TGF-β1 signaling pathways; and merely 4 compounds (2.6%) have completed *in vivo* pharmacodynamic validation. To date, no *Dendrobium* phenanthrene compound has entered clinical trials.

Thirty-seven years of research have revealed a funnel-shaped decline characterized as “rich in identification, poor in evaluation, shallow in mechanism, and scarce in vivo validation.” This systematic translational bottleneck stems from three critical gaps: (i) Selectivity and safety concerns: Current antitumor activity evaluations generally lack normal cell controls, with severe deficiency in Selectivity Index (SI) data. Most studies report only tumor cell IC_50_ values, unable to distinguish cytotoxicity from selective antitumor activity. (ii) Pharmacokinetic and druggability deficits: Systematic ADME profiling, physicochemical property analysis, and experimental druggability validation remain entirely absent, precluding assessment of oral bioavailability and drug-likeness. (iii) Mechanistic and translational gaps: Multi-omics studies, rigorous target validation, standardized *in vivo* models, and systematic toxicity evaluation are largely missing, impeding rational lead optimization.

To address these limitations, future research should prioritize: (i) Establishment of selectivity evaluation systems: Develop normal cell/tumor cell paired screening panels for early SI calculation (SI > 10 as the threshold for drug candidate selection); (ii) Druggability optimization: Leverage synthetic accessibility to develop analogs with improved ADME properties and explore formulation strategies to enhance bioavailability; (iii) In-depth mechanistic elucidation: Integrate multi-omics with CRISPR-based target validation technologies to clarify precise molecular targets; (iv) Accelerated *in vivo* validation: Utilize standardized animal models and patient-derived organoids for rapid evaluation of promising candidates; (v) Advancement of translational research: Foster interdisciplinary collaboration to advance lead compounds toward clinical trials.

*Dendrobium* phenanthrenes thus represent a paradox of potential—a structurally diverse, biologically active, and synthetically accessible compound family that remains underexploited due to systemic gaps in selectivity assessment, pharmacokinetic optimization, and translational validation. By addressing these limitations through integrated strategies, this renewable natural resource may yet fulfill its promise as a valuable scaffold for modern drug discovery against cancer, metabolic disorders, inflammatory diseases, and beyond.

## Glossary

α-SMA: α-smooth muscle actin
ABCG2: ATP binding cassette transporter G2
ADP: adenosine diphosphate
ALDH1A1: aldehyde dehydrogenase 1 family, member A1
AST: aspartate transaminase
Bax: BCL2-associated X
Bcl-2: B-cell lymphoma-2
BBB: blood-brain barrier
CAT: catalase
CCl4: carbon tetrachloride
Cd: cadmium
CDK: cyclin-dependent kinase
COL-1: type I collagen
COX-2: cyclooxygenase-2
COMP: cartilage oligometrix matrix protein
CRP: C-reactive protein
CSCs: cancer stem cells
CTGF: connective tissue growth factor
CXCL12: chemokine (C-X-C motif) ligand 12
Cytc: cytochrome C
DcR3: decoy receptor-3
DCm: transmembrane mitochondrial potential
DPPH: 1,1-diphenyl-2-picrylhydrazine
DPP-4: dipeptidyl peptidase IV
ECM: excessive extracellular matrix
eIf2α: eukaryotic initiation factor 2α
ERK1: extracellular regulated protein kinases 1
ERK2: extracellular regulated protein kinases 2
FasL: fas ligand
FT3: free triiodothyronine
FT4: free thyroxine
GDM: gestational diabetes mellitus
GLP-1: glucagon-like peptide-1
GSH-Px: glutathione peroxidase
GRP78: glucose regulated protein78 kD
GRP94: glucose regulated protein 94 kD
HSCs: hepatic stellate cells
HBD/HBA: hydrogen bond donor/acceptor counts
ICAM-1: intercellular adhesion molecule-1
**IC**_**50**_: half maximal inhibitory concentration
IkBa: nuclear factor of kappa light polypeptide gene enhancer in B cells inhibitor alpha
IKKs: IkB kinases
IL-1β: interleukin-1β
IL-6: interleukin-6
iNOS: inducible nitric oxide synthase
JNK: jun N-terminal kinase
LDH: lactose dehydrogenase
LDL: low-density lipoprotein
LPS: lipopolysaccharide
MAP: mitogen-activated protein
MAPKs: mitogen-activated protein kinases
MDA: malondialdehyde
MEK1: recombinant human mitogen activated kinase 1
MMP-2: matrix metalloproteinase-2
MMP-9: matrix metalloproteinase-9
NF-kB: nuclear factor B
OA: osteoarthritis
OA-FLS: osteoarthritis-associated fibroblast-Like synoviocytes
PEG-2: prostaglandin E₂-P
Rac1: ras-related C3 botulinum toxin substrate 1
ROS: reactive oxygen species
SOD: superoxide dismutase
SrcSTAT3-c-Myc: src-signal transducer and activator of transcription 3 (STAT3)-c-Myc
T3: triiodothyronine
T4: thyroxine hormone
TBARs: thiobarbituric acid-reactive substances
TAK1: TGFbactivated kinase 1
TGF-β1: transforming growth factor-β1
TLR4/MyD88/NF-κB: toll-like receptor 4/myeloid differentiation factor 88/nuclear factor B
TIMPs: tissue inhibitor of matrix metalloproteinases
TIMP1: tissue inhibitor of matrix metalloproteinases 1
TIMP2: tissue inhibitor of matrix metalloproteinases 2
TNF-α: tumor necrosis factor
TSH: thyroid stimulating serum thyrotropin
VCAM-1: vascular cell adhesion molecule-1
