# Supplementary material for: Structural diversity and therapeutic potential of phenanthrenes from the Dendrobium genus: a comprehensive review (1987–2025)
Source: Front Pharmacol. 2026 May 15;17:1800915. doi: 10.3389/fphar.2026.1800915 (PMC13219381; doi:10.3389/fphar.2026.1800915)
Supplement: Supplementary file 1 [file DataSheet1.docx]

**Supplementary Materials for**

**Structural Diversity and Therapeutic Potential of Phenanthrenes from the *Dendrobium* Genus: A Comprehensive Review (1987–2025)**

Jie Jian ^a^†, Junhao Wang ^a^†, Sijia Wu ^a^, Wangya Jia ^a^, Wenxu Lu ^a^, Zixu Xu ^a^, Aizheng Xiong ^a^,

Li Yang ^a^*, Hong Xu^a^*

^a^ The MOE Key Laboratory for Standardization of Chinese Medicines, The SATCM Key Laboratory for New Resources and Quality Evaluation of Chinese Medicines, Institute of Chinese Materia Medica, Shanghai University of Traditional Chinese Medicine, No. 1200 Cai Lun Road, Pudong New Area, Shanghai

† These authors contributed equally to this study.

* Corresponding author at: Institute of Chinese Materia Medica, Shanghai University of Traditional Chinese Medicine, Shanghai 201203, E-mail addresses: [xuhongtcm@shutcm.edu.cn](mailto:xuhongtcm@shutcm.edu.cn) (H. Xu).

**
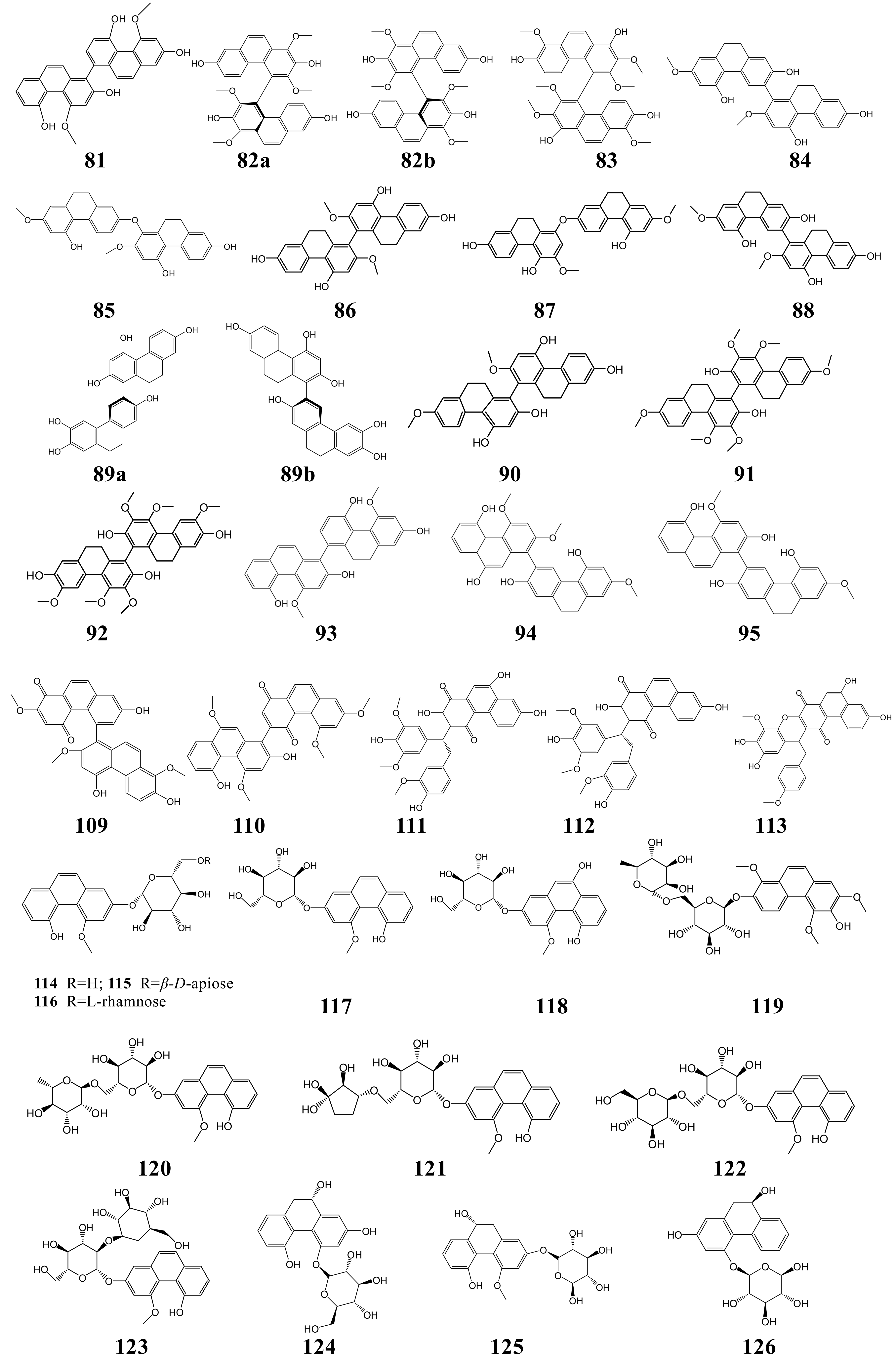
**

**Supplementary Fig. 1** Chemical structures of phenanthrene homodimers (**81-83**), dihydrophenanthrene homodimers (**84-92**), phenanthrene heterdimers (**93-95**), and phenanthraquinone dimers (**109-113**) and phenanthrene glycoside (**114-126**)isolated from the *Dendrobium* plants.

**
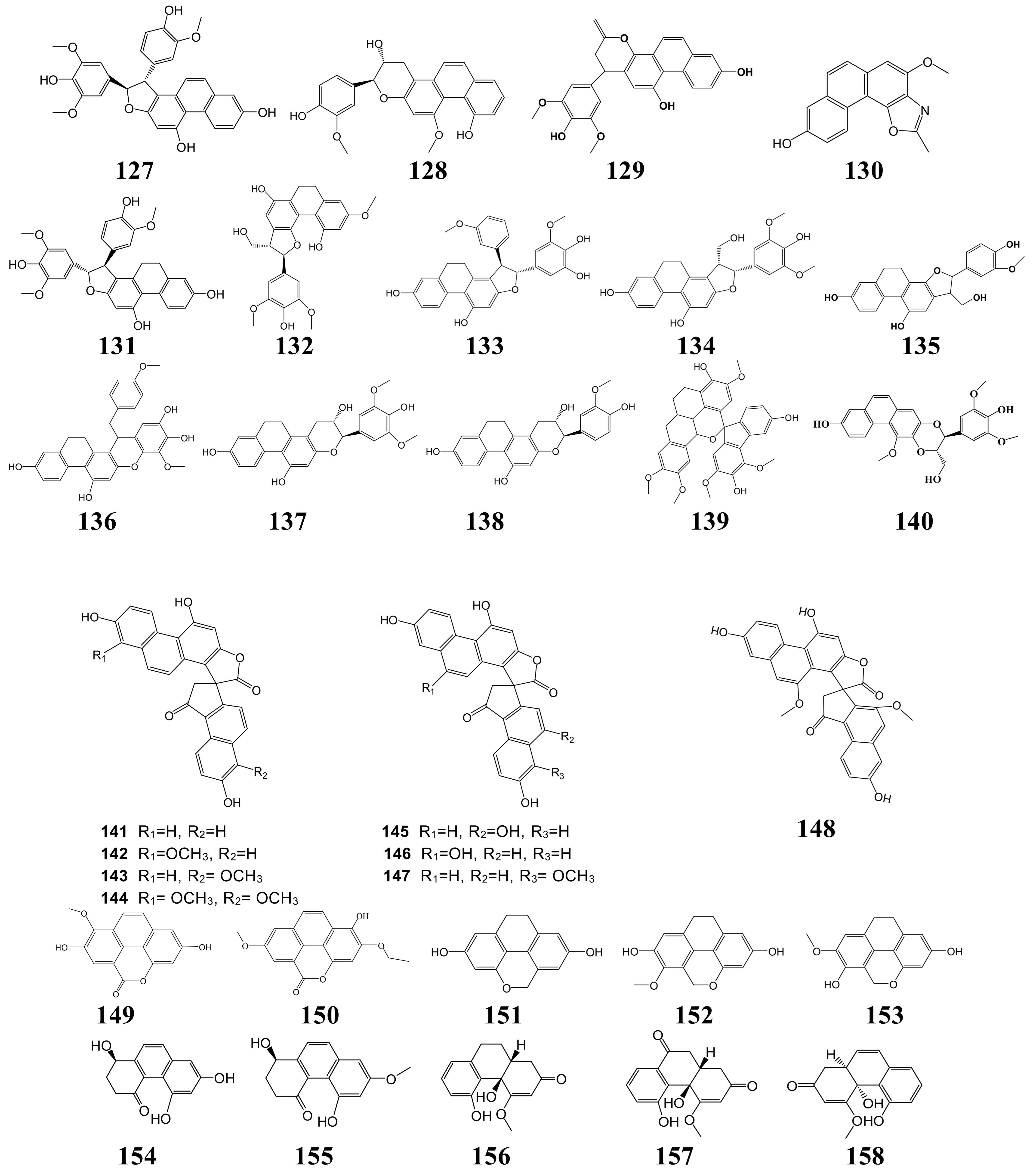
**

**Supplementary Fig. 2** Chemical structures of phenanthrene heterodimers (**127-140**),

spiro-phenanthrenes (**141-148**) and other phenanthrene derivatives (**149-158**) isolated from the *Dendrobium* plants

**Supplementary table 1.Supplementary table 1.** Statistical summary of *Dendrobium* phenanthrenes categorized by biofunctional activities (A-J. antioxidant, anti-inflammatory, cytotoxic activity, α-Glucosidase inhibition, hepatic stellate cell proliferation inhibition, antiplatelet aggregation, antiplasmodial activity, antibacterial activity, anti-neuroinflammatory activity, anti-hemin-driven LDL oxidation) and mechanistic studies ( K-O: anti-inflammatory, anti-tumor, antiplatelet aggregation, anti-fibrotic, and MAFLD amelioration).

| **NO** | **Comp.** | **A** | | **B** | **C** | **D** | **E** | **F** | **G** | **H** | **I** |  | **J** |  | **K** | **L** | **M** | **N** | **O** |
| --- | --- | --- | --- | --- | --- | --- | --- | --- | --- | --- | --- | --- | --- | --- | --- | --- | --- | --- | --- |
|  |  |  | ***In vitro* activity screening** | | | | | | | | | | |  | **Mechinism of action or *in vivo* efficiency** | | | | |
|  | **2** | + | | L1 | L1/L2 |  |  |  |  |  |  |  |  |  |  |  |  |  |  |
|  | **3** |  | | L1 |  |  |  |  |  |  |  |  |  |  |  |  |  |  |  |
|  | **6** | + | | L1/L2 |  |  |  | + |  |  |  |  |  |  |  |  |  |  |  |
|  | **8** |  | |  | L1/L2 |  |  |  |  |  | + |  |  |  |  |  |  |  |  |
|  | **9** | + | |  | L1/L2 |  |  |  |  |  |  |  |  |  |  |  |  |  |  |
|  | **10** |  | | L1 | L1/L2 |  |  |  |  |  |  |  |  |  |  | L3 |  |  |  |
|  | **11** | + | |  |  |  |  |  |  |  |  |  |  |  |  |  |  |  |  |
|  | **12** | + | |  |  |  |  |  |  |  |  |  |  |  |  |  |  |  |  |
|  | **14** |  | | L1 |  |  |  |  |  |  |  |  |  |  |  |  |  |  |  |
|  | **16** | + | | L1/L2 |  |  |  |  |  |  |  |  |  |  |  |  |  |  |  |
|  | **17** |  | | + |  |  |  |  |  |  |  |  |  |  |  |  |  |  |  |
|  | **19** |  | | L1 |  |  |  |  |  |  |  |  |  |  |  |  |  |  |  |
|  | **20** |  | | L1 |  |  |  |  |  |  |  |  |  |  |  |  |  |  |  |
|  | **21** |  | | L1 |  |  |  |  |  |  |  |  |  |  |  |  |  |  |  |
|  | **22** |  | |  | L1/L2 |  |  |  |  |  |  |  |  |  |  |  |  |  |  |
|  | **23** |  | |  | L1/L2 |  |  |  |  |  |  |  |  |  |  |  |  |  |  |
|  | **24** |  | |  | L1/L2 |  |  |  |  |  |  |  |  |  |  |  |  |  |  |
|  | **27** |  | |  |  |  | L1 |  |  |  |  |  |  |  |  |  |  | L3 |  |
|  | **31** |  | |  |  | + |  |  |  |  |  |  |  |  |  |  |  |  |  |
|  | **35** |  | | L1 |  |  |  |  |  |  |  |  |  |  |  |  |  |  |  |
|  | **36** |  | | L1 |  |  | L1 |  |  |  |  |  |  |  |  |  |  | L3 |  |
|  | **37** |  | | L1 |  |  |  |  |  |  |  |  |  |  |  |  |  |  |  |
|  | **42** |  | |  |  |  |  |  |  |  |  |  |  |  |  | L3 |  |  |  |
|  | **44** |  | | L1 |  |  |  |  |  |  |  |  |  |  |  |  |  |  |  |
|  | **45** | + | | L1/L2 | L1/L2 |  |  |  |  |  |  |  |  |  |  |  |  |  |  |
|  | **46** |  | | L1 | L1/L2 |  |  |  |  |  |  |  |  |  | + |  |  |  |  |
|  | **47** | + | | L1 | + | L1 |  | + |  |  | + |  | + |  | + | L3/L4 | L3 |  | L4 |
|  | **48** |  | |  | L1/L2 |  |  |  |  |  |  |  |  |  |  |  |  |  |  |
|  | **49** |  | | L1 |  |  | L1 |  |  |  |  |  |  |  |  |  |  |  |  |
|  | **50** |  | | L1 |  |  |  |  |  |  | + |  |  |  |  |  |  |  |  |
|  | **51** |  | |  | L1/L2 |  |  |  |  |  |  |  |  |  |  |  |  |  |  |
|  | **54** |  | |  | L1/L2 |  |  |  |  |  |  |  |  |  |  | L4 |  |  |  |
|  | **55** |  | | L1 |  |  |  |  |  |  |  |  |  |  | + |  |  |  |  |
|  | **56** |  | | L1 |  |  |  |  |  |  |  |  |  |  |  |  |  |  |  |
|  | **67** |  | | L1 |  | + |  |  |  |  |  |  |  |  |  |  |  |  |  |
|  | **71** | + | |  |  |  |  |  |  |  |  |  |  |  |  |  |  |  |  |
|  | **83** |  | |  | L1/L2 |  |  |  |  |  |  |  |  |  |  |  |  |  |  |
|  | **84** |  | |  |  |  |  |  | L1 |  |  |  |  |  |  |  |  |  |  |
|  | **85** |  | |  | L1 | + |  |  | L1 |  |  |  |  |  | + | L3 |  |  |  |
|  | **97** |  | |  | L1/L2 |  |  |  | L1 |  |  |  |  |  |  |  |  |  |  |
|  | **99** |  | |  | L1/L2 |  | L1 |  |  |  |  |  |  |  | + | L3/L4 |  | + |  |
|  | **101** |  | |  | L1 |  |  |  |  |  |  |  |  |  |  | L3 |  |  |  |
|  | **103** |  | |  | L1/L2 |  |  |  |  |  |  |  |  |  |  |  |  |  |  |
|  | **104** | + | |  |  |  |  |  |  |  |  |  |  |  |  |  |  |  |  |
|  | **105** |  | |  |  |  |  |  |  | + |  |  |  |  |  |  |  |  |  |
|  | **108** |  | |  | L1/L2 |  | + |  |  |  |  |  |  |  | + | L4 |  | L4 |  |
|  | **109** |  | |  | L1 |  |  |  |  |  |  |  |  |  |  |  |  |  |  |
|  | **111** |  | |  |  | L1 |  |  |  |  |  |  |  |  |  |  |  |  |  |
|  | **112** |  | |  |  | L1 |  |  |  |  |  |  |  |  |  |  |  |  |  |
|  | **114** |  | | L1 |  |  |  |  |  |  |  |  |  |  | + |  |  |  |  |
|  | **115** |  | | L1 |  |  |  |  |  |  |  |  |  |  |  |  |  |  |  |
|  | **116** |  | | L1 |  |  |  |  |  |  |  |  |  |  |  |  |  |  |  |
|  | **117** |  | |  | L1 |  |  |  |  |  |  |  |  |  |  |  |  |  |  |
|  | **120** |  | |  | L1 |  |  |  |  |  |  |  |  |  |  |  |  |  |  |
|  | **121** |  | |  | L1 |  |  |  |  |  |  |  |  |  |  |  |  |  |  |
|  | **124** |  | | L1 |  |  |  |  |  |  |  |  |  |  |  |  |  |  |  |
|  | **130** |  | |  | L1/L2 |  |  |  |  |  |  |  |  |  |  |  |  |  |  |
|  | **131** |  | |  |  | L1 |  |  |  |  |  |  |  |  |  |  |  |  |  |
|  | **133** |  | |  | L1 |  |  |  |  |  |  |  |  |  |  |  |  |  |  |
|  | **134** |  | |  | L1 |  |  |  |  |  |  |  |  |  |  |  |  |  |  |
|  | **138** |  | |  | L1/L2 |  |  |  |  |  |  |  |  |  |  |  |  |  |  |
|  | **139** |  | |  |  | + |  |  |  |  |  |  |  |  |  |  |  |  |  |
|  | **140** |  | |  | L1 |  |  |  |  |  |  |  |  |  |  |  |  |  |  |
|  | **149** | + | |  |  |  |  |  |  |  |  |  |  |  |  |  |  |  |  |

**"+"** *In vitro* activity screening; **L1**: Preliminary activity (single cell line, IC₅₀ < 100 μM); **L2**: Validated activity (≥2 cell lines, IC₅₀ < 50 μM, dose-dependent); **L3**: Mechanism-based activity (target engagement, pathway modulation); **L4**: *In vivo* activity (animal model verification); **L5**: Druggability (complete PK/PD profiles and safety data).
